# Proteome-Based Antigen Screening and Multi-Epitope Design Against *Cutibacterium acnes*: An In Silico Study

**DOI:** 10.3390/biology15120933

**Published:** 2026-06-15

**Authors:** Khemrutai Sripath, Teerasak E-kobon

**Affiliations:** 1 Department of Genetics, Faculty of Science, Kasetsart University, Bangkok 10900, Thailand; khemrutai.sr@ku.th; 2 Omics Center for Agriculture, Bioresources, Food and Health, Kasetsart University (OmiKU), Bangkok 10900, Thailand

**Keywords:** Acne vulgaris, *Cutibacterium acnes*, multi-epitope peptides, immunoinformatics, acne vaccine development

## Abstract

Inflammation plays a pivotal role in the pathogenesis of acne vulgaris, with *Cutibacterium acnes* recognised as a key etiological agent. The global increase in acne prevalence, coupled with the rising incidence of antibiotic-resistant strains, underscores the necessity for alternative therapeutic strategies. Vaccination has emerged as a promising approach, with various candidates targeting live-attenuated strains and specific virulence factors. Nevertheless, the expanding availability of *C. acnes* genomic data presents an opportunity to identify previously uncharacterized antigens that hold potential as novel targets for the development of next-generation acne vaccines. Therefore, this study aimed to identify core proteins among *C. acnes* genomes and evaluate their immunogenicity as potential multi-epitope peptide constructs. In addition, IA1-specific proteins of *C. acnes* were examined to develop the peptide constructs targeting acne-associated isolates. Pan-core analysis of 609 genomes identified 972 core genes. These genes were subsequently analysed for epitope prediction and antigenicity, and the highly antigenic epitopes were selected and combined for further analysis. Multi-epitope peptides were constructed based on predicted MHC-I, MHC-II, and linear B-cell epitopes, yielding four promising candidates derived from *C. acnes* core proteins and IA1-specific proteins. Molecular docking analysis indicated that both groups showed binding affinity for TLR2 and TLR4 receptors, suggesting possible molecular compatibility with these receptors. Furthermore, in silico immune simulations indicated that both types of multi-epitope peptides were associated with simulated humoral and cellular immune response profiles, although these responses require experimental validation. This computational workflow may help narrow the selection of potential acne vaccine candidates and prioritise multi-epitope peptide constructs for subsequent vaccine design steps and experimental validation.

## Simple Summary

Acne vulgaris is driven by inflammation, with *Cutibacterium acnes* playing a central role. The increasing prevalence of acne and the rise of antibiotic-resistant strains highlight the need for alternative treatments. Vaccination is a promising strategy, but many potential antigens remain unexplored despite the growing availability of *C. acnes* genomic data. In this study, we analysed 609 *C. acnes* genomes to identify 972 core genes and to evaluate their immunogenic potential. Predicted T-cell and B-cell epitopes with high antigenicity were selected to construct multi-epitope peptide candidates from both core and IA1-specific proteins. Four promising multi-epitope peptides were identified. Molecular docking suggested favourable predicted interactions with TLR2 and TLR4, and immune simulations predicted patterns consistent with potential humoral and cellular immune responses. These findings computationally prioritised multi-epitope peptide candidates for further experimental evaluation in acne-related vaccine design and development.

## 1. Introduction

Acne is a chronic inflammatory skin condition affecting up to 90% of teenagers, driven by excess sebum production, abnormal accumulation of skin cells leading to follicle obstruction, bacterial proliferation, and inflammation [1]. Worldwide, the number of acne patients has been steadily increasing, from 132 million in 1990 to 184 million in 2021 [2]. Acne impacts individuals both physically and psychologically, with research showing that it often leads to dissatisfaction with appearance, embarrassment, self-consciousness, and reduced self-confidence. These effects can impair social interactions, including with peers and the opposite gender, and may even limit employment opportunities, potentially leading to depression and suicidal thoughts [3,4,5].

*Cutibacterium acnes* (formerly known as *Propionibacterium acnes*) is a Gram-positive anaerobic bacterium that primarily resides in the sebaceous glands and hair follicles. While it plays a crucial role in maintaining skin health, it is also associated with the pathogenesis of acne vulgaris [6]. Recent studies suggest that the loss of *C. acnes* diversity may contribute to acne development, particularly through the abnormal proliferation of the IA1 *C. acnes* phylotype, which can account for up to 70% of observed phylotypes [7]. At the same time, IA2, IB, and II are decreased in inflamed acne lesions [7]. *C. acnes* plays a critical role in acne inflammation by activating the immune system. The innate immune response, which is non-specific and rapid, begins with the recognition of *C. acnes* abundance by receptors such as Toll-like receptors (TLRs), including TLR2 and TLR4, and CD14 [8]. Other receptors involved include proteinase-activated receptor-2 (PAR-2), lipid mediators, and neuropeptide receptors [8]. Following recognition, signal transduction occurs, leading to the production of immune-related factors [8]. Various pathways, such as the Toll-Like Receptor (TLR) pathway, NLRP3 Inflammasome pathway, NF-κB, and MAPK (Mitogen-Activated Protein Kinase) pathway, stimulate the production of multiple cytokines and chemokines, such as IL-1β, IL-6, IL-8, and TNF-α [8]. The adaptive immune response also contributes significantly to acne inflammation, with T-helper 1 (Th1) cells, T-helper 17 (Th17) cells, and regulatory T cells playing key roles [9].

Various acne treatments have been introduced, with the most common strategies involving systemic antibiotics and topical agents such as doxycycline, minocycline, tetracycline, and clindamycin [10,11]. Other topical treatments, including benzoyl peroxide, salicylic acid, and topical retinoids, have also been suggested for treating acne [10]. However, these treatments often cause side effects such as dryness and irritation. Moreover, antibiotic-resistant *C. acnes* strains have been observed worldwide. Misunderstandings about acne treatment, as seen in studies from Thailand and Europe [5], have contributed to the overuse of topical and systemic antibiotics, prolonged treatment courses, and the availability of over-the-counter antibiotic options, all of which contribute to the rise of antibiotic-resistant strains in acne patients [12]. These ongoing issues pose significant challenges for scientists, emphasising the urgent need for new and effective strategies to treat this widespread skin condition.

Acne vaccine development has been underway for many years. For example, the heat-killed *C. acnes* vaccine was shown to reduce inflammation (swelling) and accelerate recovery in a mouse model (22 days vs. 78 days) [13]. This vaccine reduced IL-8 production and cytotoxicity in human sebocytes, which are key markers of acne-related inflammation. However, it did not affect *C. acnes* bacterial numbers, suggesting that its action is anti-inflammatory rather than antibacterial [13]. The *C. acnes* sialidase-based vaccine represents a more targeted alternative to whole-cell-inactivated *C. acnes* vaccines, which, although quickly produced, may be less effective across diverse populations [13]. The sialidase-based vaccine, which targets sialidase (an essential enzyme for *C. acnes* adhesion to skin cells), offers greater specificity than whole-inactivated *C. acnes* vaccines, potentially increasing its effectiveness. Both vaccines reduce *C. acnes*-induced damage in human sebocytes by lowering cytotoxicity and IL-8 production; however, unlike the whole-inactivated vaccine, the sialidase vaccine also generates anti-sialidase antibodies. In mouse models, the sialidase vaccine decreased ear inflammation similarly to the whole-inactivated vaccine, but with a faster recovery time. Additionally, in an in vivo chamber model using human sebocytes implanted in mouse dermis to mimic human skin, *C. acnes* colonisation and inflammation were observed, and the sialidase vaccine significantly reduced the production of the inflammatory cytokine MIP-2 [13]. Moreover, other virulence genes may be further potential antigens of interest for an acne vaccine candidate, such as hemolysins, lipases, and sialic acid transporters [13]. In this study, we aim to use genomic and bioinformatic analyses to identify core proteins from *Cutibacterium acnes* isolates with antigenic potential, thereby enabling the design of multi-epitope peptides for acne vaccine development. Particular focus is given to targeting the IA1 phylotype of *C. acnes*, which is directly associated with acne vulgaris. Similarly, the selection of multi-epitope peptide candidates from the core genes was also used and reported in the study of the foodborne pathogen *Campylobacter jejuni* [14]. The core virulence factor (VF) genes are promising candidates for developing broad-spectrum antibiotics or vaccines that could effectively target this bacterial species [14]. Therefore, the multi-omics approach can be integrated and applied to streamline the target selection process, which is part of developing vaccines against other infectious bacteria.

## 2. Materials and Methods

### 2.1. C. acnes Genome Preparation and Genotypic Prediction Based on the PubMLST Database

Three previously prepared *C. acnes* genomes (deposited at the NCBI Genome database) were used in this study: JB7, JB13, and JB51. Other 606 *C. acnes* genomes were derived from the NCBI database, which is composed of 43 complete genomes, 7 chromosome-level, 90 scaffold-level, and 466 contig-level assembled samples (https://www.ncbi.nlm.nih.gov/datasets/genome/?taxon=1747) (accessed on 22 June 2024), and were also combined to perform further analyses.

The eight housekeeping genes following MLST8 were used to infer genotypic data, including sequence types (STs), clonal complexes (CCs), and phylotypes [15]. Classification was performed under the command line. The eight MLST gene sequences were extracted using BLAST+ (v2.13.0). A local BLAST database comprising 609 *C. acnes* genome assemblies was constructed using the makeblastdb utility from the BLAST+ suite (version 2.14.1+) [16]. Gene-specific queries were performed using command-line BLAST to identify homologs of eight housekeeping genes: *aroE*, *atpD*, *guaA*, *gmK*, *sodA*, *lepA*, *tly*, and *camp2*. Reference sequences for these genes were obtained from the PubMLST database (accessed 4 November 2024) [17]. Gene extraction was based on BLAST alignment results, with some alleles additionally retrieved by gene name from genome annotations when necessary. The extracted sequences were then searched against the PubMLST to assign the allele number, STs, CCs, and phylotypes [17].

### 2.2. Identification of Core Genes Among C. acnes Samples

Pan-core genome analysis was performed on all genomes to identify core genes, defined as those present in the majority of bacterial strains. A total of 609 genomes were downloaded via the command line and annotated using Prokka (version 1.14.6) [18]. The GFF files from all genomes were used to perform pan-core genome analysis with Roary (version 3.12.0) to identify core and accessory genes [19]. Visualisation of gene presence and absence was performed using ggplot2 (version 3.5.2) within the R environment (version 4.3.0) [20]. Core gene lists and sequences were extracted. From these, sequences corresponding to the core and soft-core gene categories were further isolated. These genes were defined as those present in 540–609 genomes, which together represent 95–99% of the total dataset. The extracted sequences were then translated into amino acid sequences using EMBOSS Transeq via the command lines [21]. The protein sequences were then predicted for localisation using PSORTb version 3.0.3 [22]. Cytoplasmic membrane, cell wall, and extracellular proteins were selected for antigenicity prediction. In addition, unique genes specific to *C. acnes* isolates from phylotype IA1 (*n* = 242, including all related CC types) were identified by comparing them with those of other phylotypes. Comparative proteome analysis was performed separately for each CC type to identify proteins unique to particular CC-defined subgroups within the IA1 lineage. Therefore, the final protein set used for epitope prediction represents CC-associated unique proteins within the phylotype IA1. These unique genes were defined as those present only in IA1 isolates and absent in other phylotypes, based on the gene presence/absence matrix generated by Roary.

### 2.3. Prediction of Linear B-Cell (LBL) Epitopes from C. acnes Core Proteins and IA1 Group-Specific Proteins

Proteins localised to the cell wall, cytoplasmic membrane, and extracellular regions were analysed for their antigenic potential. A total of nine cell wall proteins, 548 cytoplasmic membrane proteins, and 34 extracellular proteins were selected. IA1-specific proteins were also included in the analysis for this epitope prediction. Antigenicity predictions were conducted using tools available through the IEDB Analysis Resource (www.iedb.org) [23]. Predicted epitopes from BepiPred-1.0 were combined with results from additional tools, and the average scores were used to identify peptides with strong antigenicity. Additional predictors, including Emini, Chou–Fasman, Karplus–Schulz, Kolaskar–Tongaonkar, and Parker, were applied, and the integrated scores guided the selection of peptides with the highest antigenic potential [24,25,26,27,28]. Peptides were then categorized into five levels based on their average antigenicity scores, calculated from the combined results of the five prediction tools: “Very High,” “High,” “Medium,” “Low,” and “Very Low,” corresponding respectively to the top 5%, 80–95%, 60–80%, 40–60%, and below the 40th percentile. Peptides in the “Very High” group were designated as having the highest antigenicity. In addition, peptide length was limited to 6–25 amino acids, a common range for B-cell epitopes [29,30]. Peptides that met these criteria were selected for further validation, including predictive assessments of antigenicity, toxicity, and immunogenicity using additional tools such as VaxiJen v2.0, AllergenOnline, and ToxinPred [31,32,33]. Epitopes predicted to be antigenic, non-toxic, non-allergenic, and with high antigenicity scores were selected. Among the cytoplasmic membrane-predicted epitopes, the largest group, the ten peptides with the highest antigenicity scores (as predicted by VaxiJen v2.0), were selected for further analysis.

Conservancy analysis of the predicted linear B-cell epitopes (LBLs) was performed to evaluate their conservation across different genomes using the Epitope Conservancy Analysis tool on the IEDB server (http://tools.iedb.org/conservancy/, accessed on 10 December 2024) [34]. Only epitopes with a conservancy greater than 80% were considered. The conservancy analysis followed these main steps: to obtain the relevant protein sequences, BLAST searches were conducted against the NCBI protein reference sequence database using the amino acid sequences of the target genes. The resulting BLAST hits provided matching protein sequences from *C. acnes* isolates, which were then used to evaluate the conservation of each predicted epitope across different genomes.

### 2.4. Cytotoxic T Lymphocyte (CTL) Epitope Prediction from C. acnes Core Proteins and IA1 Group-Specific Proteins

CTL epitopes from core *C. acnes* proteins localised to the cell wall, cytoplasmic membrane, extracellular region, and IA1-specific proteins were predicted using the NetCTL-1.2 server [35]. Default parameters were used, including a weight of 0.15 for C-terminal cleavage, 0.05 for TAP transport efficiency, and an epitope identification threshold of 0.75. HLA class I supertypes provided by the server were used in the prediction, covering A1, A2, A3, A24, A26, B7, B8, B27, and B58. Subsequently, the MHC class I binding alleles for each predicted epitope were identified using the MHC I binding module within the IEDB (Immune Epitope Database) server (2009-09-01 version) (http://tools.immuneepitope.org/mhci/, accessed on 10 December 2024) [34]. Three prediction methods were employed: average relative binding (ARB), artificial neural network (ANN), and stabilised matrix method (SMM) [34,36,37]. All available HLA alleles were utilised in this step, totalling 40 different alleles. The results from the NetCTL-1.2 server and the MHC-binding predictions were then combined for each HLA class I supertype. To select the best candidate CTL epitopes, the peptide sequence must meet two criteria: (1) it must be predicted as an epitope by the NetCTL-1.2 server, and (2) it must exhibit an IC50 value less than 500 nM in the MHC-binding predictions. Finally, the predicted epitopes were assessed for antigenicity, allergenicity, and toxicity. These evaluations were performed using VaxiJen v2.0 [31], AllergenOnline [32], and ToxinPred [33], respectively. The selected epitopes were then subjected to epitope conservation analysis to validate their conservation across different antigens. This step was done using the epitope conservation analysis tool on the IEDB server, following the same procedure as for LBL identification [34]. The conservancy percentage must exceed 80% for the compared sequences. Epitopes demonstrating high antigenicity and broad population coverage were selected for vaccine construction.

### 2.5. Identification of Major Histocompatibility Complex (MHC) Class II Binding Molecules from C. acnes Core Proteins and IA1 Group-Specific Proteins (HTL)

Three groups of localised proteins were analysed to predict MHC class II binding peptides using tools from the IEDB Analysis Resource. Predictions were based on 15-mer peptides, consistent with typical MHC-II binding lengths. A reference set of seven human HLA alleles from the IEDB database was used. NetMHCIIpan EL 4.1 predicted potential epitopes, while NetMHCIIpan BA 4.1 assessed binding affinity [38,39]. Peptides with the lowest IC50 values and percentile ranks were initially selected and further evaluated for binding across additional HLA alleles. Peptides were retained if they showed strong binding (IC50 < 500 nM, percentile rank ≤ 10) and were then assessed for antigenicity, allergenicity, and toxicity using VaxiJen [31], AllergenOnline [32], and ToxinPred [33]. Only those that were highly antigenic, non-toxic, non-allergenic, and capable of binding multiple HLA alleles with high affinity (IC50 < 50 nM, percentile rank ≤ 2) were selected. Their predictive ability to induce IFN-γ, IL-4, and IL-10 was evaluated using the IFNepitope [40], IL4pred [41], and IL10pred [42] tools, applying relevant threshold scores. Peptides predicted to induce all three cytokines were prioritised. Final candidates were analysed for population coverage and sequence conservation, and only those conserved in at least 80% of the compared sequences were selected for further consideration.

### 2.6. Human Homology Analysis of the Predicted CTL and HTL Epitopes from C. acnes Core Proteins and IA1 Group-Specific Proteins

All predicted peptides that met the selection criteria for both CTL and HTL epitopes were evaluated for human homology to ensure their safety for application. This analysis was conducted using BLASTp (protein BLAST) against the non-redundant protein sequence database on the NCBI platform, with *Homo sapiens* (taxid: 9606) specified as the reference organism and an E-value threshold of 0.05 [43]. Epitopes that showed no significant homology to human sequences, as indicated by E-values below the threshold, were prioritised for epitope development.

### 2.7. Prediction of CTL and HTL Epitope Structures and Molecular Docking Against HLA Antigens

Both CTL- and HTL-epitope-predicted peptides were modelled in 3D using the PEP-FOLD v3.5 server, utilising 200 simulations and the sOPEP sorting model prior to docking [44]. Docking simulations were then carried out between the selected peptides and two widely distributed alleles: HLA-A01:01, representing MHC class I, and HLA-DRB101:01, representing MHC class II. The corresponding PDB structures for these alleles (IDs: 6AT9 and 1QEW) were retrieved from the RCSB Protein Data Bank [45,46]. ClusPro 2.0 was used to dock the CTL and HTL epitopes to the MHC molecules, with the CTL and HTL epitopes assigned as ligands and the MHC molecules as receptors [47]. Docked complexes were then evaluated based on energy scores and the number of cluster members; lower energy scores and higher numbers of clustered members suggest greater consistency and reliability, as multiple docking simulations converge on similar binding positions. The interactions between the epitope peptides and Toll-like receptors were analysed and illustrated using PDBsum and Chimera X [48,49].

### 2.8. Population Coverage Analysis of the Predicted CTL and HTL Epitopes

The predicted HTL and CTL epitopes were computationally assessed for their population coverage, which estimates the proportion of individuals likely to respond to a specific set of epitopes based on recognised MHC restrictions. This analysis was performed using the population coverage analysis tool from the IEDB analysis resources (http://tools.iedb.org/population/, accessed on 17 May 2025), covering the global population, sixteen geographic regions, and Thailand [50]. Population coverage was calculated separately for CTL and HTL epitopes, corresponding to MHC class I and class II, respectively.

### 2.9. Construction of Predicted Multi-Epitopes and Computational Characterisation of Their Features

The multi-epitope peptide was constructed by sequentially combining the predicted CTL, HTL, and LBL epitopes. The first group of multi-epitopes included epitopes selected from all core predictions. Another set of multi-epitopes was constructed using only IA-1-specific epitopes, incorporating all relevant sequences. To enhance stability and facilitate immune responses, linker sequences were introduced between each epitope type: the AAY linker (Ala-Ala-Tyr) for CTL epitopes, the GPGPG linker (Gly-Pro-Gly-Pro-Gly) for HTL epitopes, and the KK linker (Lys-Lys) for LBL epitopes. Additionally, an adjuvant was attached to the N-terminus of the multi-epitope construct using the EAAAK linker (Glu-Ala-Ala-Ala-Lys) to further strengthen simulated immune responses. These modifications were applied to both multi-epitope groups. For the multi-epitope design, CTL, HTL, and LBL epitopes with high predicted antigenicity were selected. The predicted multi-epitope combinations were generated randomly using a Python script version 3.11.5. This script created three separate lists for CTL, HTL, and LBL epitopes, which were shuffled within each group to ensure variability. Epitope sequences from these lists were then sequentially joined to generate all possible combinations to explore alternative epitope arrangements. The combined epitope constructs were analysed for their physicochemical properties, including the number of amino acids, molecular weight, theoretical pI, total number of negatively charged residues (Asp + Glu), total number of positively charged residues (Arg + Lys), molecular formula, estimated half-life, instability index, aliphatic index, and grand average of hydropathicity (GRAVY). These properties were predicted using the ProtParam tool available on the ExPASy proteomics server [51]. The predicted antigenicity of the multi-epitope constructs was assessed using VaxiJen v2.0 with the default threshold (0.4) [31], while predicted allergenicity was evaluated using Allertop v2.1 [52], AllergenFP v1.1 [52], and AlgPred v2.0 [53]. The presence of transmembrane helices and potential signal peptides within the constructs was determined using the TMHMM v2.0 and SignalP 4.1 servers [54,55]. Human homology analysis of the multi-epitope constructs was performed using BLASTp against the non-redundant protein sequence database of *Homo sapiens* (taxid: 9606). Two algorithms were employed for this analysis: BLASTp (protein–protein BLAST) and PSI-BLAST (Position-Specific Iterated BLAST) [43]. To ensure safety, the multi-epitope constructs were required to exhibit no similarity to human proteins. Homologous sequences to human proteins (E-value < 0.05) were excluded, and the PSI-BLAST algorithm was set to prevent percentage identity from exceeding 40%. Finally, multi-epitope constructs with high predicted antigenicity, non-allergenic properties, zero to one transmembrane helix, and no homologous sequences to human proteins were selected for further validation.

The multi-epitopes used in the constructs, along with their corresponding HLA alleles (Class I and Class II), were submitted to the population coverage analysis tool of the IEDB database (http://tools.iedb.org/population/, accessed on 17 May 2025) to evaluate the coverage of the multi-epitopes, using the combined Class I and Class II calculation option (Supplementary Tables S5 and S6). The same geographical regions and countries used in the previous population coverage assessment of individual epitopes were applied in this analysis.

### 2.10. Secondary and Tertiary Structure Prediction of Multi-Epitope Constructs

Both predicted multi-epitope constructs, the core *C. acnes* protein construct and the IA1-specific protein construct, were assessed for their secondary structure using two online tools: SOPMA and NetSurfP-2.0 [56,57]. NetSurfP-2.0 was used to generate secondary structure illustrations of the constructed multi-epitope peptides, while SOPMA predicted the number of amino acids forming each secondary structure type, including alpha helices, beta turns, and random coils. The default SOPMA parameters were applied, including four conformational states (helix, sheet, turn, coil) and a similarity threshold of eight. Tertiary structure prediction of the multi-epitope constructs was performed using Robetta, which employs deep-learning-based methods such as RoseTTAFold and TrRosetta for homology modelling and structural constraint integration [58]. Five predictive models were generated, and the best one was selected based on in silico structural assessments. Procheck assessed model quality through Ramachandran plots and G-factors, evaluating dihedral angles, residue geometry, and stereochemical reliability [59]. Favoured regions suggest stability, while disallowed regions and low G-factors indicate potential errors [59]. Predicted model quality was further validated using the Z-score from ProSA-Web (https://prosa.services.came.sbg.ac.at/prosa.php, accessed on 21 February 2025), which provides a quantitative measure of structural alignment with experimentally determined proteins. A Z-score within the range typical for native proteins of similar size indicates a properly modelled structure, while significant deviations may suggest modelling errors [60]. Additional model validation was conducted using the SAVES v6.1 server (https://saves.mbi.ucla.edu/), which integrates tools such as ERRAT, for analysing non-bonded atomic interactions (with lower error values indicating higher structural quality), and Verify3D, which evaluates the compatibility between the model’s 3D structure and its 1D amino acid sequence [61,62].

Furthermore, MolProbity (https://molprobity.biochem.duke.edu/) was used to assess in silico atomic interactions and structural geometry. This step included analysis of clashscore (to detect steric clashes), rotamer quality (to identify favoured versus poor side-chain conformations), Ramachandran outliers (for unfavourable backbone dihedral angles), and the Rama Z-score (to compare ϕ-ψ angle distributions with high-quality protein structures). Structural geometry was further evaluated through Cβ deviations (greater than 0.25 Å indicating backbone distortion), along with assessments of bond lengths and bond angles [63]. All validation results were reviewed collectively to select the most reliable and representative predicted structural model.

### 2.11. Molecular Docking of Multi-Epitope Constructs with Toll-Like Receptor Molecules

Because Toll-like receptors (TLR-2 and TLR-4) are involved in innate immune recognition of *C. acnes*, molecular docking was performed as a preliminary in silico approach to assess potential molecular compatibility between the predicted multi-epitope constructs and TLR-2/TLR-4. The crystal structures of TLR-2 (PDB ID: 2Z7X) and TLR-4 (PDB ID: 3FXI) were used for this analysis [64,65]. Prior to docking, the TLR structures were prepared by removing ligands and water molecules, adding hydrogen atoms, and assigning appropriate charges. The most suitable predicted multi-epitope construct models were used as ligands, and docking was performed with ClusPro 2.0, using TLR-2 and TLR-4 as receptors [47]. The docked model exhibiting the lowest binding energy and the highest number of clustered members was selected, as lower energy indicates stronger binding, and a higher cluster count suggests consistency across multiple docking simulations. The resulting receptor–ligand interactions were analysed and visualised using PDBsum and ChimeraX [48,49].

### 2.12. Molecular Dynamics Analysis of TLR–Epitope Construct Complexes

The complexes formed between TLR receptors and multi-epitope constructs identified in the previous docking analysis were further analysed using molecular dynamics to assess their predicted stability and flexibility. iMODS (Internal Coordinates Normal Mode Analysis Server) (https://imods.iqf.csic.es/) was employed for this purpose [66]. iMODS is a web-based tool that performs normal mode analysis (NMA), a simplified, rapid alternative to full-scale molecular dynamics simulations, to evaluate the dynamic behaviour of protein complexes computationally [66]. The molecular dynamics/NMA analysis was conducted using the coarse-grained Cα elastic network model (CA-model), with a 15 Å interaction cutoff, and 20 normal modes were calculated. The docked complex structures were submitted to iMODS, and key parameters, including deformability, B-factor, eigenvalues, covariance matrices, and elastic networks, were analysed to characterise the predictive structural flexibility and stability of the complexes.

### 2.13. In Silico Simulation of Immune-Response Profiles of Multi-Epitope Constructs

Eight predicted multi-epitope constructs were evaluated for their predicted capability to generate simulated immune response profiles using the online simulation tool C-ImmSim (https://kraken.iac.rm.cnr.it/C-IMMSIM/, accessed on 22 May 2025) [67]. This tool utilises amino acid sequences to simulate immune interactions computationally. All settings were kept at their default values, including a random seed of 1234 and a simulation volume of 10, except for the injection schedule. In silico injections were administered at time-steps 1, 84, and 170, corresponding to four-week intervals (with each time-step representing 8 h in real time, and time-step 1 representing the initial injection at time = 0) [68]. The simulation ran for a total of 1050 time-steps.

### 2.14. In Silico Codon Optimisation and Cloning of the Predicted Multi-Epitope Constructs

The initial expression construct was designed using *Escherichia coli* K12 as the host strain. Codon optimisation was performed using the JCat (Java Codon Adaptation Tool) online server (https://www.jcat.de/), with *E. coli* K12 selected as the target organism [69]. Additional parameters were enabled to avoid rho-independent transcription terminators and prokaryotic ribosome binding sites. To facilitate downstream cloning, recognition sites for the restriction enzymes BamHI and XhoI were excluded during the optimisation process. After optimisation, BamHI and XhoI recognition sequences were added to the 5′ and 3′ ends of the DNA sequence, respectively, to create compatible cloning sites. The pET-28a(+) plasmid was chosen as the expression vector, and in silico cloning was performed using SnapGene software (version 8.0.3) (www.snapgene.com). The optimised insert was positioned between the BamHI and XhoI sites within the vector’s multiple cloning site (MCS), allowing directional cloning and maintaining the correct reading frame under the control of the T7 promoter. The final constructs were verified for proper orientation, in-frame insertion, and the presence of a C-terminal His-tag for downstream protein purification.

### 2.15. Summary of Criteria for Protein Prioritisation, Epitope Peptide Selection, and Multi-Epitope Construct Design

The initial protein pool was narrowed by selecting proteins predicted to be localised in the cytoplasmic membrane, cell wall, or extracellular region, as these proteins are more likely to be exposed to host immune recognition (Step 2). The selected proteins were then prioritised using multiple computational criteria, including predicted antigenicity, non-allergenicity, non-toxicity, epitope conservancy, MHC-binding potential, cytokine-inducing potential, population coverage, and absence of significant human homology (Steps 3–8). For the IA1-related analysis, candidate proteins were derived from CC-associated unique proteins within the IA1 lineage, rather than from all IA1 genomes collectively (Step 2). The final protein candidates were therefore selected for the design based on a combination of criteria relevant to computational epitope peptide selection, including simulated immune accessibility, predicted antigenic potential, safety-related filtering, conservation, HLA-binding breadth, population coverage, and suitability for multi-epitope construct design (Steps 9–13). This computational prioritisation was a strategy to narrow candidate targets for future experimental validation, rather than as definitive evidence of biological efficacy.

## 3. Results

### 3.1. Genotypic Identification and Classification of C. acnes Based on Genome Analysis

**Figure 1:**
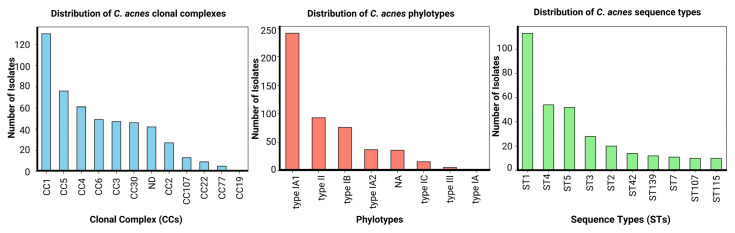
MLST allelic profile classification of the *C. acnes* isolates used in this study.

Of the 609 genomes analysed, three genomes: JB7, JB13, and JB51, were derived from a previous study and belonged to sequence types (STs) ST5, ST36, and ST213; clonal complexes (CCs) CC5, CC2, and CC172; and phylotypes IB, IA2, and IA1, respectively. Among the remaining 606 genomes (excluding Thai strain genomes), the most prevalent ST, CC, and phylotype were ST1, CC1, and phylotype IA1, respectively (Figure 1). Some *C. acnes* genomes could not be assigned MLST profiles because they lacked one or more MLST8 alleles. In such cases, assignments were made based on the closest matching profiles.

The classification is based on the MLST8 scheme and current data from the PubMLST database (accessed on 4 November 2024). The bar chart uses different colours to represent distinct data types shown on the *X*-axis: blue for clonal complexes (CCs), orange for phylotypes, and green for sequence types (STs). The *Y*-axis indicates the number of *C. acnes* isolates. The ST chart includes only the ten most prevalent isolates.

### 3.2. Pan-Core Analysis of 609 C. acnes Genomes

**Figure 2:**
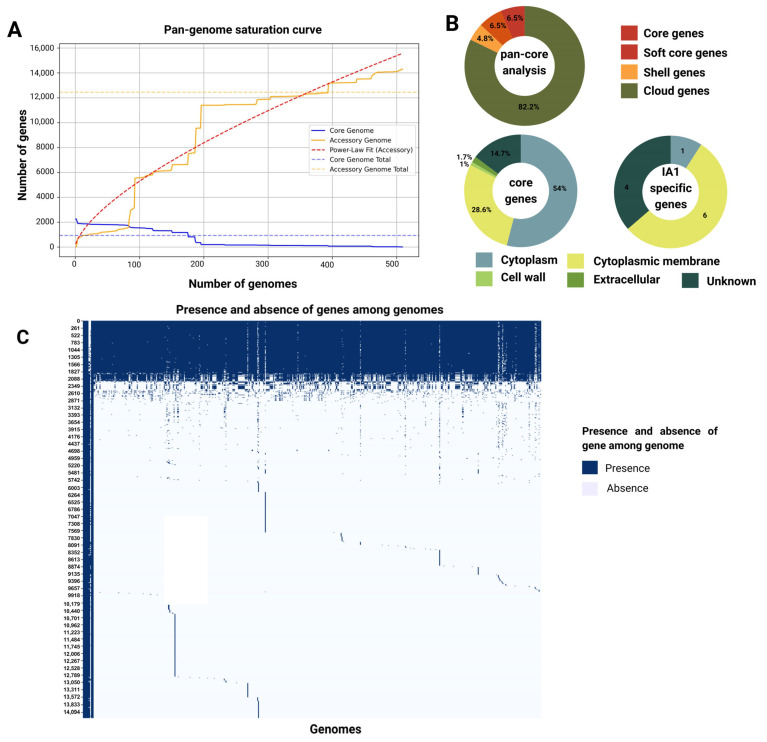
Pan-core analysis of 609 *C. acnes* isolates. (**A**) Pan-genome saturation curve showing the number of core (blue) and accessory (orange) genes as genomes are added. Dotted lines indicate estimated total core and accessory genome sizes; the red dashed line represents a power-law fit for accessory genes. (**B**) Pie charts showing pan-genome composition (**top**), subcellular localisation of core genes (**bottom-left**), and IA1-specific genes (**bottom-right**). Most core genes are cytoplasmic, while IA1-specific genes are more variably distributed. (**C**) Presence–absence matrix of gene clusters across genomes. Rows represent genes, columns represent genomes. Dark blue indicates gene presence, and light blue indicates absence.

Pan-core analysis of all genomes revealed a total of 14,306 genes, of which 972 were identified as core genes. An additional 936 genes were classified as soft core genes, present in at least 95% of the genomes. Meanwhile, 689 genes were designated as shell genes, occurring in approximately 50% of genomes, and the remaining 11,754 genes were categorised as cloud genes, found in only approximately 15% of genomes (Figure 2B,C). A saturation curve was generated to observe the trend in core gene prediction. It showed that the number of core genes stabilised between 200 and 500 genomes, suggesting that this number of genomes is sufficient to represent *C. acnes* diversity (Figure 2A).

In contrast, the number of accessory genes, including shell and cloud genes, gradually increased between 200 and 500 genomes and continued to rise beyond this range (Figure 2A). This trend followed a power-law distribution, indicating that as more genomes are included, the number of accessory genes will likely continue to increase. However, this study primarily focused on the core genes. A total of 1907 core genes, comprising both core and soft core genes, were extracted from the pan-core analysis and translated into amino acid sequences. Among the resulting proteins, 1035 were localised to the cytoplasm, 548 to the cytoplasmic membrane, 33 to the extracellular space, and 9 to the cell wall, while 282 proteins had unknown localisation (Figure 2B).

To identify genes potentially specific to IA1-type *C. acnes*, which is associated with acne vulgaris, gene presence was analysed across 609 genomes. Of these, 242 genomes were classified as IA1. Six genes were found in 44–55 IA1 genomes, and another five genes were found in 13–15 IA1 genomes (Supplementary Table S1). These genes and their predicted localisations of encoded proteins include amino acid permease (GadC) (cytoplasmic membrane), regulatory protein ArsR (cytoplasm), triacylglycerol lipase (unknown localisation), cadmium resistance transporter (cytoplasmic membrane), and seven hypothetical proteins (three with unknown localisation and four in the cytoplasmic membrane) (Figure 2B). The *gadC* gene was identified in 54 out of 61 *C. acnes* isolates belonging to clonal complex CC4, representing 89% of CC4 isolates. Additionally, five hypothetical proteins were found in 72–87% of CC4 isolates. In contrast, *cmtR* and four other hypothetical protein-encoding genes were detected in CC1, CC3, and CC172 but at lower frequencies, accounting for only 7.3% of isolates across these clonal complexes. Proteins specifically identified in IA1-type *C. acnes* (CC1, CC3, CC172, and CC4-associated) were subsequently prioritised for antigenicity analysis as potential targets for IA1-specific vaccine development.

### 3.3. Linear B-Cell Epitope Prediction from C. acnes Core Proteins

**Figure 3:**
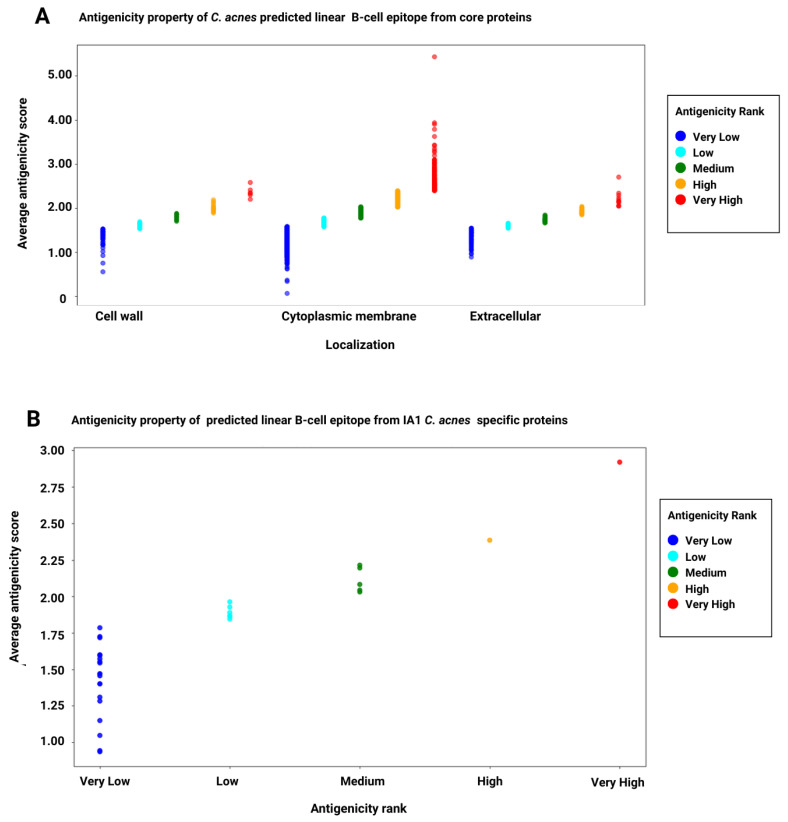
Antigenicity of predicted linear B-cell epitopes from *C. acnes* core proteins (**A**) and proteins specific to IA1 *C. acnes* (**B**). The X axis shows subcellular localisation (**A**) and antigenicity rank (**B**). The antigenicity scores are on the *Y*-axis and classified into five categories based on percentile ranges: very high ((**top**) 5%), high (80–95%), medium (60–80%), low (40–60%), and very low ((**below**) 40%). Each group is visually distinguished by uniquely coloured points corresponding to its antigenicity level. The average antigenicity scores of epitopes are derived from five prediction tools (as described in the Materials and Methods section).

Four localised proteins, specifically those associated with the cell wall, cytoplasmic membrane, and extracellular space, were selected to predict their antigenic properties using the integrated IEDB tools (Supplementary Table S2). Among cell wall-localised peptides that met the selected length criteria, 2 exhibited very high antigenicity, 4 showed high antigenicity, 15 were medium, 21 were low, and 62 displayed very low antigenicity (Figure 3). In the extracellular compartment, 11 peptides were predicted to have very high antigenicity, 33 high, 43 medium, 43 low, and 87 very low antigenicity. For peptides derived from cytoplasmic membrane-localised proteins, 168 exhibited very high antigenicity, 504 high, 672 medium, 671 low, and 1344 very low antigenicity. In total, 181 peptides with very high antigenicity were identified across all analysed proteins. Twenty-one very high predicted average scores were selected as representatives of core-predicted LBL epitopes. The antigenicity predicted by VaxiJen of the 21 predicted epitopes was approximately 2.14 ± 0.78 (Supplementary Table S3).

Eleven type IA1 *C. acnes*-specific proteins were predicted to contain B-cell epitopes. Among these, one peptide showed very high potential, followed by 1, 9, 12, and 40 peptides with high, medium, low, and very low potential, respectively (Figure 3). The peptides with very high and high antigenicity were derived from the cadmium resistance transporter (CadD) and amino acid permease (GadC), respectively. However, since these two candidates showed similarity to known allergens, the focus shifted to epitopes with medium antigenicity. Consequently, two IA1-specific proteins, an ArsR/SmtB family transcription factor (CmtR) and a hypothetical protein (Group4654), were identified as having medium antigenicity based on average predicted scores—VaxiJen 2.0 antigenicity predictions of 1.7389 and 1.1529, respectively (Supplementary Table S3). The three epitopes with the highest antigenicity were selected for the construction of core-predicted multi-epitope peptides: LBL1, LBL2, and LBL3. From the IA1-specific group, two predicted epitopes, LBL22 and LBL23, were utilised.

### 3.4. Cytotoxic T Cell (CTL) MHC-I Epitope Prediction of C. acnes Core Proteins and IA1 Group-Specific Proteins

Following CTL epitope prediction, peptides from each localised protein were assessed using stringent predicted criteria, including high antigenicity, non-allergenicity, and non-toxicity. Seven CTL epitopes predicted from *C. acnes* core proteins were selected for high antigenicity scores, including five epitopes localised to the cytoplasmic membrane (CTL1–CTL5), one from the cell wall (CTL6), and one from extracellular proteins (CTL7) (Supplementary Table S4). Additionally, two *C. acnes*-specific epitopes, derived from the amino acid permease protein (CTL8 and CTL9), satisfied these selection criteria (Supplementary Table S4). The binding affinity of these epitopes to MHC-I molecules was computationally evaluated, with the majority demonstrating strong predicted binding scores, as suggested by predicted IC50 values below 500 nM across multiple MHC alleles (Supplementary Table S5). The predicted IC50 (half-maximal inhibitory concentration) in the context of MHC-I binding prediction refers to the proposed concentration of a peptide (in nanomolar, nM) required to inhibit 50% of the binding of a standard peptide to a specific MHC class I molecule. Conservancy analysis revealed high conservation of the predicted epitopes across *C. acnes* isolates, underscoring their putative potential as multi-epitope peptide targets. Furthermore, all nine CTL epitopes were suggested to be non-homologous to human proteins, with E-values below 0.05, mitigating concerns regarding potential autoimmune responses. Population coverage analysis indicated generally low coverage percentages across most populations (Supplementary Figure S1). However, selected epitopes, including CTL1, CTL2, and CTL3, exhibited significantly higher global coverage rates of 70.2%, 62.1%, and 55.3%, respectively (Supplementary Figure S1). Notably, CTL3 achieved the highest predicted coverage in the Thai population at 75.6%, highlighting its potential relevance for region-specific epitope development (Supplementary Figure S1).

### 3.5. Prediction of Helper T-Cell (HTL) MHC-II Epitopes from C. acnes Core and IA1 Group-Specific Proteins

T-helper epitope prediction for core proteins localised to the cell wall, extracellular space, and cytoplasmic membrane identified 23, 23, and 336 candidate sequences with predicted high antigenicity, respectively. These peptides were further evaluated computationally based on additional criteria. A total of twenty peptides met all predefined selection criteria, all of which originated from proteins localised to the cytoplasmic membrane (Supplementary Table S6). These peptides were subsequently analysed for sequence conservation across the *C. acnes* species, with six (HTL1-HTL6) showing sequence identities of 90% or higher (Supplementary Table S7). Two proteins derived from the IA1-specific protein also met the predefined criteria, designated HTL7 and HTL8. The in silico MHC-II binding activities of the identified epitopes were subsequently evaluated (Supplementary Figure S2). MHC alleles with predicted IC50 values less than 500 nM were considered indicative of strong epitope-MHC binding.

### 3.6. Prediction of CTL and HTL Epitope Structures and Binding to the MHC Molecules

**Figure 4:**
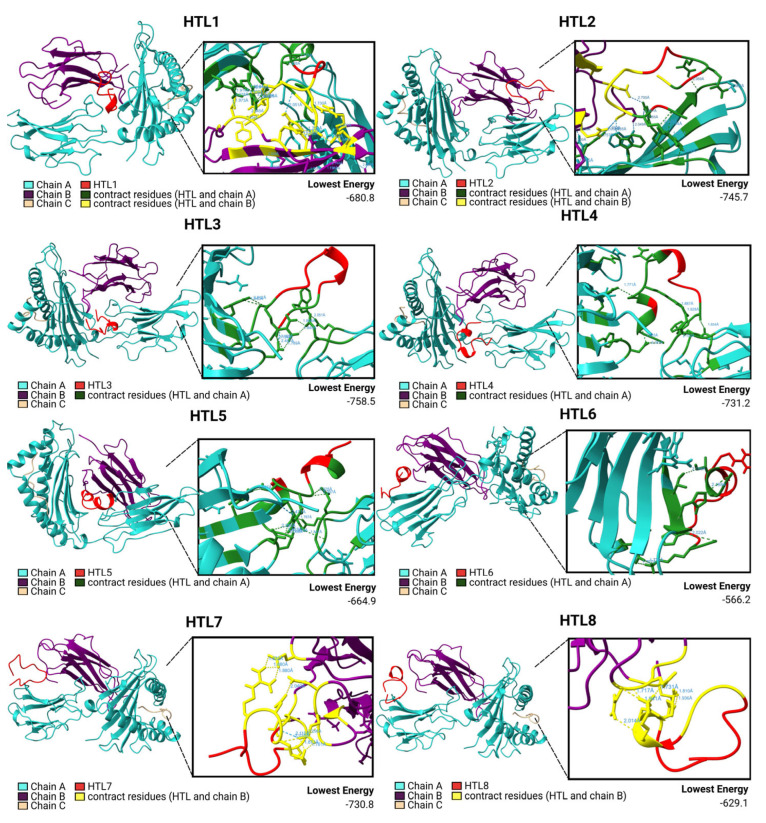
Molecular docking of *C. acnes*-derived HTL epitopes with the HLA-DRB1*01:01 MHC-II molecule using ClusPro.

**Figure 5:**
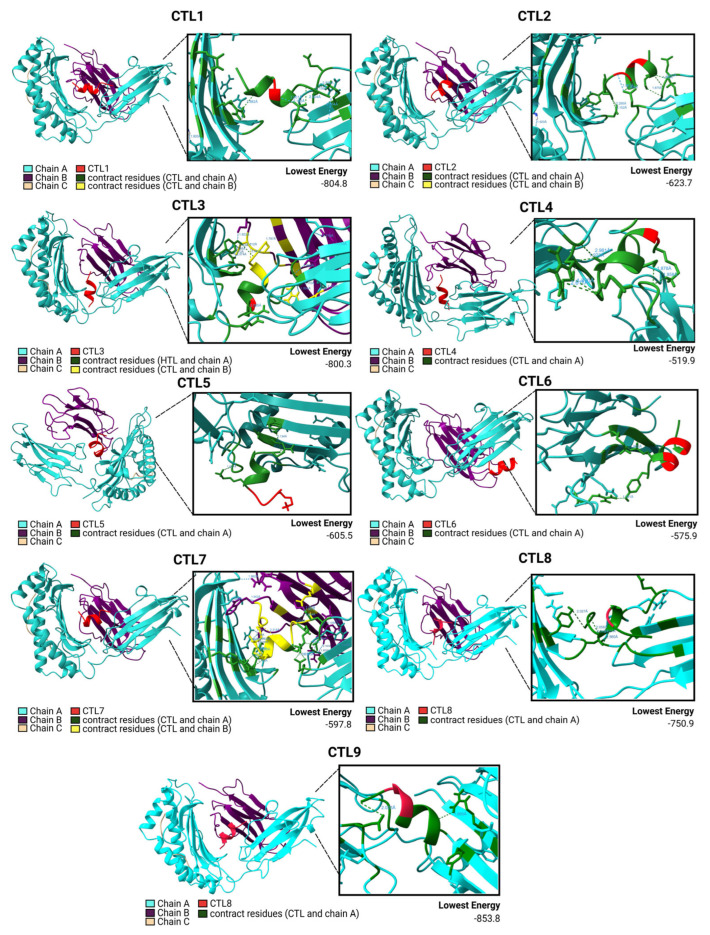
Molecular docking of *C. acnes*-derived CTL epitopes with the HLA-A*01:01 MHC-I molecule using ClusPro.

Binding interactions between the eight predicted HTL epitopes and MHC-II molecules were computationally evaluated via ClusPro docking against the HLA-DRB1*01:01 allele (Figure 4). HTL3 exhibited the most favourable predicted binding energy of –758.5 kcal/mol, indicating the strongest interaction; HTL2, HTL4, HTL7, and HTL1 followed in descending order. Overall, the eight HTL epitopes exhibited an average lowest binding energy of –688.4 ± 62.1 kcal/mol in silico (Supplementary Table S8). The average cluster size, defined as the number of docked poses grouped by RMSD into the same cluster, was 243.0 ± 67.8 members, suggesting consistent docking solutions for these HTL–MHC II complexes (Supplementary Table S8). Similarly, nine predicted CTL epitopes were docked against the HLA-A*01:01 allele (Figure 5), yielding an average lowest binding energy of –681.4 ± 114.1 kcal/mol; CTL9, CTL1, and CTL3 exhibited the strongest predicted affinities, respectively. The average cluster size for these CTL–MHC I complexes was 436.0 ± 105.5 members, suggesting consistent computational docking outcomes (Supplementary Table S8).

Each of the eight panels (HTL1–HTL8) shows the global HTL–MHC-II complex on the left and a magnified view of the predicted peptide–MHC binding interface on the right. In all views, the MHC α-chain (Chain A) is colored cyan, the MHC β-chain (Chain B) is purple, and the invariant chain (Chain C) is beige. The docked HTL epitope is shown in red. Residues forming intermolecular contacts between the epitope and Chain A are highlighted in green, while contacts with Chain B are highlighted in yellow. Below each inset, the lowest docking energy (in kcal/mol) for that epitope–MHC complex is indicated. These recurrent clusters of low-energy poses demonstrate the predicted binding strength and reproducibility of each HTL epitope’s interaction with HLA-DRB1*01:01.

Each panel (CTL1–CTL9) shows the predicted full protein–peptide complex (left) and a close-up of the peptide–MHC binding interface (right inset). In all views, the MHC heavy chain is colored cyan (Chain A), the β_2_-microglobulin or second MHC chain is purple (Chain B or C, as labelled), and the docked CTL epitope is red. Residues participating in intermolecular contacts are highlighted in green (epitope and MHC) and yellow (where two MHC chains interact). The lowest binding energy for each epitope–MHC complex (in kcal/mol) is indicated beneath the inset.

### 3.7. Multi-Epitope Construction and Selection

All possible multi-epitope constructs were generated by shuffling the predicted high-immunogenicity epitopes. Ten epitopes were utilised in the core predicted constructs, including CTL1, CTL2, CTL3, CTL4, HTL1, HTL2, HTL3, LBL1, LBL2, and LBL3, while CTL8, CTL9, HTL7, HTL8, LBL22, and LBL23 were used for the IA1-specific constructs. Among the core constructs, four multi-epitope peptide candidates, MULTI437, MULTI443, MULTI797, and MULTI803, were selected based on the highest predicted antigenicity scores, with an average antigenicity score of 1.4438 (Supplementary Table S9). For the IA1-specific constructs, all potential candidates, including IA1-3, IA1-4, IA1-7, and IA1-8, were retained for further validation, with an average antigenicity score of 1.18 (Supplementary Table S9).

Despite using the same predicted CTL, HTL, and LBL epitopes, all candidates shared identical values for several physicochemical properties. These included the number of amino acid residues, molecular weight, theoretical isoelectric point (pI), the total number of negatively charged residues (Asp + Glu), positively charged residues (Arg + Lys), molecular formula, estimated half-life, and grand average of hydropathicity (GRAVY). The shared properties (average values) of core predicted constructs were as follows: 147 amino acid residues, a molecular weight of 15,754.26 Da, a theoretical pI of 9.93, 10 negatively charged residues, 20 positively charged residues, and a molecular formula of C_720_H_1116_N_202_O_189_S_4_. The GRAVY score was −0.070, and the aliphatic index was 81.70%. Although the instability index showed slight variation, it averaged approximately 26.68, indicating a predicted high level of stability across all constructs. One transmembrane helix was observed in each of the four constructs, while no signal peptide sequences were identified. The four predicted IA1-specific epitope constructs exhibited the following average values: molecular weight of 10,868.59 Da, theoretical pI of 9.99, 10 negatively charged residues, 16 positively charged residues, a molecular formula of C_494_H_772_N_146_O_128_S_2_, and a GRAVY score of −0.260. The aliphatic index was 79.90%, and the average instability index was 19.91, also suggesting favourable predicted stability. Additionally, the IA1-specific constructs contained a single transmembrane helix but lacked signal peptides. The estimated half-lives of both constructs were 1 h in mammalian reticulocytes (in vitro), 30 min in yeast (in vivo), and more than 10 h in *Escherichia coli* (in vivo). None of the constructs shared homology with human proteins.

The population coverage of the combined epitopes was also evaluated (Supplementary Figure S3). The core-predicted constructs showed 96.7% global coverage, while the IA1-specific constructs demonstrated 87.24% coverage. In the Thai population, the coverage reached 91.37% for the core constructs and 76.1% for the IA1-specific constructs. Across other countries, the core-predicted multi-epitopes showed consistently high coverage, averaging approximately 89.54% ± 10.98%, whereas the IA1-specific constructs had lower coverage, averaging 77.27% ± 15.03%.

### 3.8. Secondary and Tertiary Structure of Predicted Multi-Epitope Constructs

**Figure 6:**
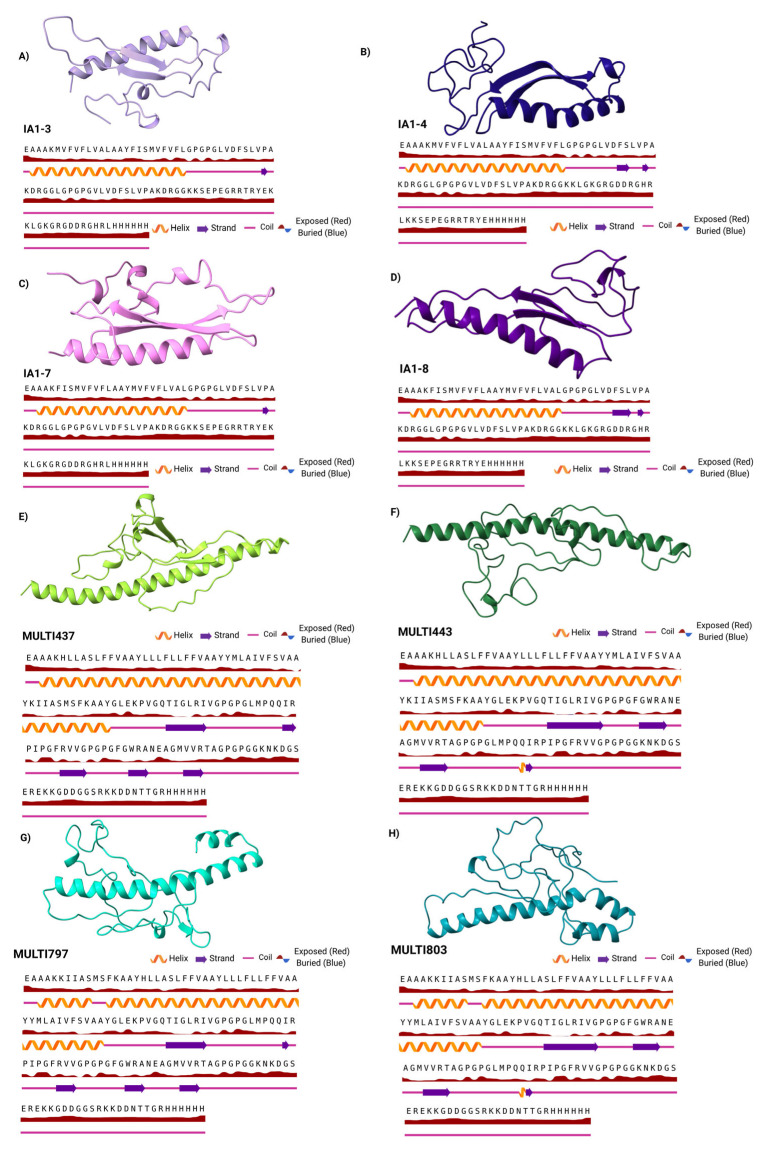
Predicted 3D structures and secondary structure elements of IA1 and MULTI multi-epitope constructs. Panels (**A**–**D**) display the predicted tertiary and secondary structures of the IA1 constructs (IA1-3, IA1-4, IA1-7, IA1-8), while panels (**E**–**H**) depict the corresponding structures for the MULTI constructs (MULTI437, MULTI443, MULTI797, MULTI803). Each structure includes a ribbon diagram showing the 3D conformation and a linear schematic representation of the secondary structure elements aligned with the amino acid sequence. Secondary structure elements are colour-coded: alpha-helices (orange), beta-strands (purple), and coils (pink). Residue exposure is indicated below the secondary structure: red bars represent exposed residues, and blue triangles represent buried residues.

The secondary structure predictions for both multi-epitope construct groups showed similar distributions within each group (Figure 6). In the core *C. acnes* multi-epitope constructs, comprising 147 amino acids, the majority of residues were predicted to be exposed structural elements. Specifically, the predicted secondary structure comprised 36.05% alpha-helices, 7.82% beta turns, 44.22% random coils, and 11.90% extended strands. For the IA1-predicted structure, the composition included 23.00% alpha-helices, 12.00% beta turns, 41.00% random coils, and 24.00% extended strands. These results indicate that both multi-epitope construct groups predominantly consist of random coils, followed by alpha-helices, extended strands, and beta turns. Notably, both structures exhibited a high proportion of exposed residues rather than buried ones. This predominance of exposed random coils and alpha helices suggests increased flexibility and potential structural stability, which may support predicted epitope accessibility, pending experimental confirmation.

All predicted multi-epitope constructs were assessed for their 3D structures and overall quality. Each construct was initially modelled in Robetta, yielding five models per construct. The best model for each construct was selected based on predefined quality criteria. Structural quality was evaluated using Ramachandran plot statistics and G-factors (Supplementary Table S10). The core-predicted structures showed 96.40% ± 1.73% of residues in the most favoured regions, 0.00% in disallowed regions, and an average G-factor of 0.34 ± 0.01 (Supplementary Table S10). In comparison, the IA1-specific structures showed 95.38% ± 0.75% in favoured regions, 0.00% in disallowed regions, and an average G-factor of 0.29 ± 0.03 (Supplementary Table S10). Additional model selection parameters included the Z-score (overall model quality), the ERRAT score (an overall quality factor), the Verify3D score, and clashscore. The core-predicted multi-epitope constructs had average values of −4.77 ± 0.26 (Z-score), 83.71 ± 7.74 (ERRAT), 61.40% ± 1.95% (Verify3D), and 0.61 ± 0.02 (clashscore). The IA1-specific multi-epitope constructs exhibited values of −4.44 ± 0.33, 93.78 ± 1.92, 71.75% ± 4.32%, and 0.72 ± 0.04, respectively, for the same parameters. These metrics suggest that all representative models were of acceptable predicted structural quality. The selected models were subsequently used to assess their Toll-like receptor-binding ability via molecular docking simulations.

### 3.9. Molecular Docking and Dynamic Simulation of Multi-Epitope Constructs and Toll-Like Receptors

**Figure 7:**
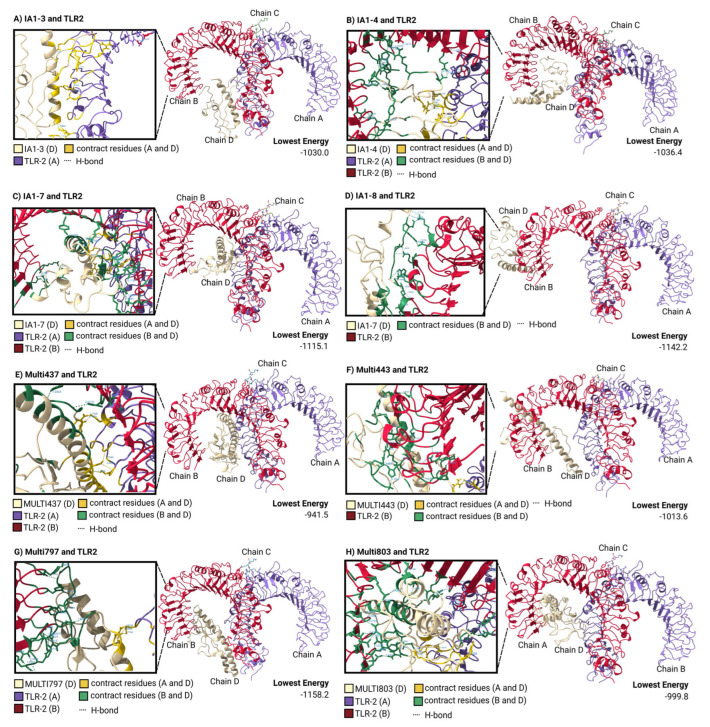
Molecular docking of IA1 and MULTI multi-epitope constructs with TLR2 receptor. Panels A–H illustrate the molecular docking interactions between various epitope constructs and the Toll-like receptor 2 (TLR2). Each panel shows the docked complex in two views: an overview of the full docking model (**right**) and a magnified view of the interface highlighting contact residues (**left**). Panels (**A**–**D**) depict IA1 constructs, and Panels (**E**–**H**) depict MULTI constructs docked with TLR2. Each docking interaction is labelled with its predicted lowest binding energy (in kcal/mol).

**Figure 8:**
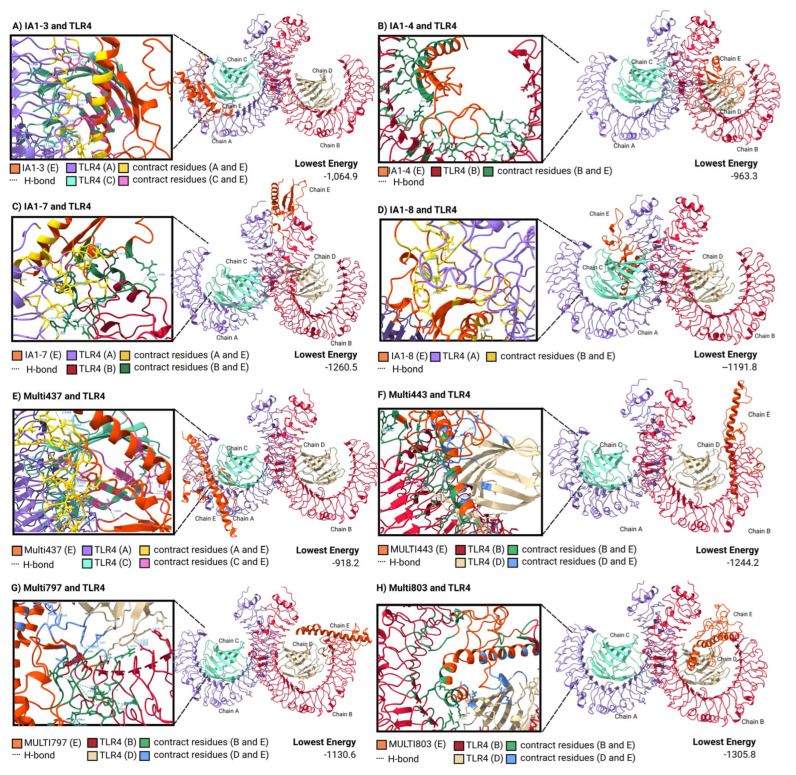
Molecular docking of IA1 and MULTI multi-epitope constructs with TLR4 receptor. Panels (**A**–**H**) illustrate the molecular docking interactions between various epitope constructs and the Toll-like receptor 4 (TLR4). Each panel shows the docked complex in two views: an overview of the full docking model (**right**) and a magnified view of the interface highlighting contact residues (**left**). Panels (**A**–**D**) depict IA1 constructs, and Panels (**E**–**H**) depict MULTI constructs docked with TLR4. Each docking interaction is labelled with its lowest predicted binding energy (in kcal/mol).

Protein docking between the multi-epitope constructs and Toll-like receptors (TLRs) was performed to assess the predicted binding affinity of the constructs to TLRs, which are involved in innate immune recognition. The IA1-specific-predicted multi-epitopes showed mean lowest predicted binding energy values of −1080.93 ± 56.26 kcal/mol with TLR2 and −1120.13 ± 132.33 kcal/mol with TLR4, along with an average number of cluster members of 100.5 ± 65.00 and 66.25 ± 19.75, respectively (Figure 7 and Figure 8). Meanwhile, the core-predicted multi-epitopes exhibited even lower predicted energy scores of −1028.28 ± 92.05 kcal/mol (TLR2) and −1149.70 ± 170.49 kcal/mol (TLR4), suggesting favourable predicted docking affinity (Figure 7 and Figure 8). These were associated with average cluster member counts of 67.75 ± 22.19 and 59.25 ± 23.61, respectively (Supplementary Table S11). Overall, both multi-epitope constructs showed favourable predicted binding affinities for TLR receptors, and core-predicted multi-epitopes showed more favourable predicted interaction scores with TLR4 than IA1 clusters, as indicated by predicted energy scores (Supplementary Table S11).

Moreover, in silico binding interactions between the constructs and TLR molecules were analysed. The interactions between the multi-epitope constructs and TLR2 exhibited variable numbers of bonds, with an average of 18.38 ± 6.54 hydrogen bonds, 4.63 ± 2.64 salt bridges, and 218.50 ± 80.89 non-bonded contacts (Supplementary Table S11). However, IA1-7, IA1-8, MULTI797, and MULTI803 displayed significantly higher numbers of hydrogen bonds (24, 28, 22, and 21, respectively), suggesting more favourable predicted interface formation. In contrast, IA1-3, IA1-4, MULTI437, and MULTI443 exhibited lower hydrogen bond numbers. The average number of salt bridges across most complexes was approximately 4, except for IA1-3, which did not form any. Additionally, non-bonded contacts, including van der Waals and hydrophobic interactions, were most common in constructs such as IA8. The predicted interaction between constructs and TLR-4 also suggested variable bonding and showed higher numbers of salt bridges, hydrogen bonds, and non-bonded contacts, with averages of 4.75 ± 3.34, 17.50 ± 7.45, and 222.25 ± 51.06, respectively (Supplementary Table S11). The MULTI437 contained the highest number of total hydrogen bonds, followed by IA1-7 and IA1-4. The docking analysis revealed that both multi-epitope groups exhibited predicted stable interactions with Toll-like receptors. MULTI797 exhibited the most favourable predicted binding energy when docked with TLR2, reaching as low as −1158.2 kcal/mol, and was associated with a high cluster size of 90 members. For the IA1-specific multi-epitope construct, the most favourable predicted binding energy was −1142.20 kcal/mol, with a cluster size of 197 members. These binding energies were accompanied by extensive hydrogen bonding and consistent salt-bridge formation, indicating computationally favourable receptor–construct interfaces. Docking of TLR4 with the epitope constructs suggested that MULTI803, MULTI443, IA1-7, and IA1-8 exhibited the most favourable predicted binding energies at −1244.2, −1305.8, −1260.5, and −1191.8 kcal/mol, respectively. These constructs also showed high cluster sizes of 95, 50, 85, and 68 members, which corresponded with extensive bond interactions in their complexes.

Molecular dynamics of the docked complexes were analysed to assess their stability and flexibility. Deformability and B-factor analyses indicated that both the IA1 and MULTI epitope constructs could computationally form stable complexes with TLR2 and TLR4 (Supplementary Figures S4 and S5). The deformability plots showed low flexibility across most regions, with only minor peaks observed at loop or terminal residues. Similarly, B-factor values remained low to moderate, suggesting limited predicted atomic fluctuation in the simulated complexes. The close alignment between the NMA-derived and experimental PDB B-factors provided computational support for the docked models. Additionally, the eigenvalue plots displayed a gradual increase, beginning with relatively low initial eigenvalues. This pattern suggests a computationally favourable dynamic profile while maintaining overall structural rigidity. The predicted motions were mainly captured by a few dominant modes (Supplementary Figures S6 and S7). Furthermore, cross-correlation and atomic displacement analyses for both epitope types and receptors showed predicted correlated motions across residue pairs, reflecting coordinated domain dynamics. Low predicted atomic fluctuations in key regions further supported the simulated structural rigidity of the docked complexes (Supplementary Figures S8 and S9).

### 3.10. Assessment of Immunological Simulation of Predicted Epitope Constructs

The simulation of immune response events following administration of the designed multi-epitope peptides was conducted to predict the immunogenic potential of the epitope constructs over a simulated 350 days. The simulation results also revealed distinct patterns in antibody production following simulated antigen administration (Supplementary Figure S10). Shortly after each antigen injection, antigen levels are predicted to peak sharply and then decline rapidly, consistent with simulation patterns of antigen recognition. Predicted antibody responses follow a characteristic kinetic pattern: IgM levels rise quickly, peaking early during the primary response phase, while IgG1 and IgG2 levels increase more gradually, becoming more prominent after the second and third exposures (Supplementary Figure S10). These predicted responses, including delayed but sustained IgG production, suggest class switching and predicted memory B cell-like activation profiles. The combined antibody response (IgM + IgG) illustrates the predicted cumulative effect of repeated antigen exposures, often showing higher peaks with each subsequent dose (Supplementary Figure S10). Examining individual multi-epitope constructs, IA1-3 and IA1-7 were associated with stronger predicted IgM responses relative to IgG, suggesting a more primary immune profile with limited class switching.

In contrast, IA1-4 and IA1-8 showed a dominant predicted IgG1 + IgG2 response, exceeding IgM levels after the third antigen exposure. This result was consistent with a simulated adaptive memory-like profile and predicted class-switched antibody profiles. Among the *C. acnes* core-predicted constructs, MULTI437, MULTI443, and MULTI797 were consistently associated with robust predicted IgG responses, with IgG1 and IgG2 levels clearly surpassing IgM levels. In contrast, MULTI803 exhibited approximately equal levels of IgG and IgM, suggesting a less pronounced class-switched response. This simulated response pattern supported their prioritisation for future immunogenicity testing. The simulation of cytokine and interleukin concentrations provides valuable predictive insights into the immune response profiles associated with the multi-epitope constructs (Supplementary Figure S11). Across all tested constructs, both *C. acnes* IA1-specific multi-epitopes (IA1-3, IA1-4, IA1-7, IA1-8) and *C. acnes* core-predicted multi-epitopes (MULTI257, MULTI443, MULTI797, MULTI803) showed a consistent simulated immune-response pattern. Peak concentrations of IFN-γ, IL-2, and IL-10 occurred rapidly following each antigen exposure, indicating a simulated cytokine-response pattern. Notably, elevated levels of IFN-γ and IL-2 were consistent with a predicted Th1-skewed response profile. The predictive presence of IL-10 may reflect regulatory feedback mechanisms within a Th1-polarised environment. The absence of Th2-associated cytokines, such as IL-4, further suggests that these constructs were associated with a simulated Th1-skewed immune-response profile, which may warrant further experimental assessment of cellular immune responses.

Predicted B-cell activation induced by the multi-epitope constructs was also analysed. All constructs were associated with a simulated rapid increase in the total B cell population at each post-vaccination time point, after which levels remained stable (Supplementary Figure S12). Overall, immunological simulation of the multi-epitope constructs showed a predicted level of memory B cells that gradually decreased and persisted at around 100 cells/mm^3^ after the simulated 350 days. T-helper (TH) cell dynamics simulated over 350 days revealed distinct responses across the multi-epitope constructs. All candidates showed predicted primary and booster response peaks, with total TH cells peaking shortly after each simulated injection. Memory TH cells were predicted to emerge following the initial response and to decline gradually, suggesting a memory-like response profile. Among IA1 constructs, IA1-8 was predicted to induce the highest TH response and memory cell persistence, while IA1-7 showed the weakest. MULTI constructs were generally predicted to outperform IA1-specific constructs, with MULTI257 exhibiting the strongest and most sustained memory TH cell response computationally. These predictive results highlight more favourable simulated T-helper response profiles of IA1-8 and MULTI257. The immune simulation results indicated that all epitope constructs were associated with predicted balanced immune-response profiles.

### 3.11. Codon Optimisation and In Silico Vector Construction of Multi-Epitope Constructs

The vector construction of the predicted multi-epitope candidates was designed for subsequent cloning and evaluation of their immunogenic potential. Codon optimisation of the construct sequences was performed, and *Escherichia coli* (strain K12) was selected as the host for preliminary expression analysis. The codon adaptation index (CAI) of the optimised sequences indicated a high level of adaptation to the host, with average CAI values of 1.0 and 0.94 for the IA1-specific and core-predicted constructs, respectively, suggesting efficient expression potential. Additionally, the GC content of the optimised sequences was appropriate for *E. coli*, with values of approximately 53.67% and 54.20% for the IA1-specific and core-predicted constructs, respectively. The optimised sequences were subsequently inserted into the pET-28a(+) vector using BamHI and XhoI restriction sites. The final constructs had total sizes of 5776 bp and 5635 bp for the core-predicted and IA1-specific constructs, respectively, with insert sizes of 312 bp and 456 bp (Supplementary Figure S13).

## 4. Discussion

Acne vulgaris is one of the most common skin conditions worldwide, affecting individuals both physically and psychologically. *Cutibacterium acnes* plays a significant role in this disorder by aggravating its severity and inducing pain through inflammation. It activates the skin’s immune system, triggering the release of inflammatory molecules and the recruitment of immune cells, which contribute to the swelling, redness, and pus characteristic of acne [9]. Therefore, targeting inflammation may represent an effective therapeutic strategy for managing acne vulgaris.

In this study, the core proteins of *C. acnes*, predicted through pan-core genome analysis, were investigated to identify representative and conserved targets. These core proteins may represent computationally prioritised targets for further investigation. The pan-core analysis of 609 genomes identified 972 core genes, which are present in approximately 99% of the genomes. According to the power-law fit, this number of core genes stabilised as the number of genomes compared increased, suggesting that these core genes are good representatives of *C. acnes* genomes. The number of identified core genes is consistent with the previous finding by Cobian et al. (2021), which compared 255 genomes and identified 1194 core genes [70]. Differences between *C. acnes* phylotypes may reflect distinct pathogenic properties. Phylotype I has been more strongly associated with acne vulgaris compared to phylotype II [71]. Within phylotype I, the IA1 subgroup is most consistently linked to acne, as it is frequently detected in acne lesions, whereas other phylotypes are often absent [15,72]. In contrast, other phylotypes, such as IB, II, and III, are generally not associated with acne but have been linked to soft-tissue and medical-implant infections [15,73]. Although type IC has also been implicated in acne pathogenesis, supporting evidence remains limited, and its representation in the current study was relatively low [15]. Consequently, this study focused primarily on IA1 strains. Of the 609 genomes, 242 were IA1 *C. acnes*; therefore, genes present only in some IA1 *C. acnes* groups were the focus. These genes included seven hypothetical protein-encoding genes and four genes with known functions. Amino acid permease, Cd(II)/Pb(II)-sensing metalloregulatory transcriptional regulator CmtR, Bifunctional helix-turn-helix transcriptional regulator/GNAT family N-acetyltransferase, and Cadmium resistance transporter were found exclusively in IA1 phylotype *C. acnes*. The IA1 phylotype of *C. acnes* has been suggested to be associated with acne following overgrowth, whereas other phylotypes, such as IA2, IB, IC, II, and III, are less prevalent in acne lesions [74]. Beyond the phylotype level, specific clonal complexes have been implicated in acne pathogenesis, with certain complexes, such as CC1, CC3, and CC4, showing a stronger association with acne than others [15]. The glutamate/gamma-aminobutyrate antiporter (GadC) and four hypothetical proteins were identified in most CC4 *C. acnes* genomes, occurring in approximately 53 of 61 genomes. The *gadC* gene encodes a transporter that exchanges extracellular glutamate (Glu) for intracellular γ-aminobutyric acid (GABA), a process that helps remove protons from the cytoplasm and contributes to acid resistance. This mechanism has been well described in food-borne hemorrhagic *Escherichia coli* [75]. Homologs of *gadC* have also been identified in other bacterial species, including lactic acid bacteria (*Lactiplantibacillus plantarum*, *Lactococcus lactis*, *Levilactobacillus brevis*, *Limosilactobacillus fermentum*, and *Limosilactobacillus reuteri*) [76] and *Enterococcus* spp. [77], *Brucella* spp. and *Shigella* spp. [78], where it functions as part of the glutamate decarboxylase (GAD) system that enables survival in acidic environments. Human skin typically maintains a pH range of 4 to 6, creating a challenging environment for microbial survival [79]. Although the function of this gene in *C. acnes* has not yet been experimentally validated, its presence in clonal complex 4 (CC4) may suggest an adaptive advantage in such acidic conditions. The specific presence of the other four hypothetical proteins in CC4 may suggest an adaptive advantage, warranting further validation to elucidate their functional roles. Moreover, the cadmium resistance transporter and the Cd(II)/Pb(II)-sensing metalloregulatory transcriptional regulator CmtR were found only in CC1, CC3, and CC172. These genes were predominantly found in *C. acnes* ST115, a member of clonal complex 3 (CC3), with 8 out of 10 isolates carrying them. The remaining five isolates of *cmtR* harbouring IA1 included four from ST1 (CC1) and one from ST213 (CC172) (Supplementary Table S1). Cadmium (Cd^2+^) is a heavy metal known to disrupt cellular processes. The cadmium resistance transporter CadD functions as an efflux pump, enabling bacteria to resist cadmium toxicity by exporting Cd^2+^ ions out of the cell [80]. In addition, the *cmtR* gene is involved in heavy metal homeostasis by regulating gene expression in response to toxic metal ions, such as cadmium. This gene has been identified in *Mycobacterium tuberculosis*, where it functions as a cadmium-responsive regulator [81]. Similarly, in *Acidithiobacillus ferrooxidans*, upregulation of *cmtR* under high-cadmium conditions supports its role in heavy-metal resistance [82]. This finding raises the question of why *C. acnes* harbours these genes. Acne vulgaris is a chronic skin disorder with multifactorial causes. Notably, a study by Ikaraoha et al. (2017) reported significantly elevated blood levels of cadmium and lead (Pb) in Nigerian individuals with acne, with concentrations increasing progressively from mild to severe cases [83]. These findings suggest a potential role for cadmium resistance mechanisms in *C. acnes* during acne pathogenesis. Further investigation is warranted to confirm the presence and function of these genes in *C. acnes* and to explore their association with acne vulgaris, which may provide new insights into the disease’s pathogenesis and guide the development of novel treatments.

Multi-epitope vaccines have emerged as a promising strategy to stimulate both cellular and humoral immune responses, as supported by immunoinformatic studies [84]. Several multi-epitope vaccines have been proposed as treatments against various pathogens, including viruses such as SARS-CoV-2 (COVID-19) [85] and porcine epidemic diarrhoea virus (PEDV) [86], as well as bacteria such as *Haemophilus influenzae* [87] and *Yersinia pestis* [88]. However, no multi-epitope peptide predictions for *Cutibacterium acnes* have been reported to date. Therefore, this study aimed to construct multi-epitope peptides based on core and IA1-specific proteins of *C. acnes*. Core genes localised to surface-exposed regions were translated into amino acid sequences and used to predict epitopes. Multi-epitope peptide constructs were then designed using linear B-cell (LBL), cytotoxic T-cell (CTL), and helper T-cell (HTL) epitopes selected for their immunogenic potential. The predicted epitopes showed antigenicity scores above the defined threshold, indicating prioritisation by antigenicity-prediction criteria. The constructs were not selected solely on predicted antigenicity; they were further evaluated using multiple in silico criteria, including antigenicity, allergenicity, physicochemical properties, solubility, structural stability, and immune simulation profiles. These properties were also considered alongside previously published, experimentally validated epitope constructs, providing supporting references for the potential relevance of the designed candidates. The epitope ordering may influence antigen processing, presentation, folding, and linker accessibility, and the current shuffling-based approach should be interpreted as a computational prioritisation step. Future experimental validation and rational optimisation will be required to confirm whether the selected epitope arrangements are biologically meaningful.

The epitope adjuvant sequence was computationally added to the N-terminal of the construct using an EAAAK linker to support construct design based on commonly used immunoinformatic strategies [84]. Linkers, including AAY, GPGPG, and KK, were incorporated to enhance the structural integrity and immunogenicity of the epitope constructs; AAY and GPGPG linkers improved CTL and HTL epitope processing, respectively, while KK linkers facilitated the separation of B-cell epitopes, enhancing solubility, flexibility, and antigen processing via cathepsin B, ensuring individual peptide presentation without eliciting unwanted antibody responses [89,90]. All possible combinations of candidate epitopes were computationally explored to select the most predicted antigenic peptides. The properties of the epitope constructs were evaluated, revealing predicted sizes of approximately 100 and 147 amino acids for the IA1-specific and core protein-based epitopes, respectively. These lengths are notably shorter than those reported in previous studies, where construct sizes typically ranged from 200 to 300 amino acids [91,92,93]. Despite the smaller size, the constructs proposed in this study demonstrated high predicted antigenicity, with average scores of 1.4438 for core-based constructs and 1.17645 for IA1-specific constructs, supporting their prioritisation based on predicted antigenicity. These values exceed those reported in previous studies, which typically range from 0.6 to 0.8 [94,95,96]. This finding suggests high predicted antigenicity; however, the simulated immune-response profiles require experimental validation, including receptor-binding assays, reporter cell assays, cytokine profiling, and immune-cell activation studies. Therefore, the docking findings provided supportive computational evidence for prioritisation, rather than confirmation of biological activity.

The negative GRAVY scores suggest hydrophilic properties that may support aqueous compatibility, consistent with previous reports of multi-epitope designs [97,98]. Moreover, the proposed epitope constructs had favourable predicted stability-related parameters, as indicated by instability indices below 40. The population coverage of both vaccine constructs was high, at 96.7% for the core-predicted construct and 87.24% for the IA1-specific construct. However, regional analysis revealed that South Africa exhibited the lowest population coverage for both vaccines. The finding may be attributed to biased HLA allele representation in the IEDB, where approximately half of the dataset originates from European and American populations, as also discussed in the previous study [99]. Focusing on the Thai population, the core-predicted multi-epitopes demonstrated high coverage, whereas the IA1-specific epitopes showed moderate coverage. Further adjustments may be necessary to improve the Thai-specific coverage of IA1-based epitopes.

ClusPro was used for protein–protein docking to predict stable interactions between molecules by simulating their binding and ranking models based on cluster size and docking energies, including both the lowest-energy and the cluster’s core energy [47]. Docking between the designed epitopes and TLR molecules yielded predicted low-energy scores and large cluster sizes, consistent with favourable binding conformations between the two molecules, as recommended by ClusPro [100]. The binding interface revealed hydrogen bonds, salt bridges, and non-bonded contacts, indicating predicted stable interactions between the epitopes and TLR molecules. Notably, hydrogen bonds involving buried or partially buried donors and acceptors are known to enhance the stability of protein complexes significantly [101]. In addition, salt bridges and other non-bonded interactions also play important roles in maintaining protein stability [102,103]. The molecular dynamics simulation results indicated that the docked complexes maintained predicted structural stability, with no significant atomic distortions in the multi-epitope constructs. This finding was supported by low predicted values observed in the deformability, B-factor, and eigenvalue analyses, consistent with reports from the previous multi-epitope design in *Helicobacter pylori* [104]. Biochemical characterisation will be required to validate the predicted interaction data further.

The in silico immunological simulation suggested that all constructs would be rapidly recognised by the immune system, with predicted early increases in antibody levels—primarily IgM, followed by IgG. IA1-4, IA1-8, MULTI437, and MULTI797 were associated with strong predicted IgG responses, whereas other constructs produced predominantly IgM. Therefore, these simulation results may be consistent with predicted immunoglobulin class switching, a critical process that generates functional diversity in the humoral immune response [105]. In terms of cytokine production, all constructs were predicted to elevate the cytokine levels following antigen exposure. IFN-γ was predicted to increase sharply, likely driven by Th1 cells producing innate cytokines such as IL-12 and IL-18. These cytokines further promote the activation of monocytes and macrophages, as well as the development of cytotoxic T lymphocytes (CTLs), which play a key role in cellular immunity [106,107]. In this simulation, predicted IL-12 production was observed during the first simulated vaccination period. Moreover, the observed early peak in IL-2 expression corresponds with its well-established role in promoting the proliferation and differentiation of naïve T helper cells during the primary immune response [108]. B cells, which are essential players in the adaptive immune response, recognise antigens and differentiate into plasma cells that produce antibodies or memory B cells that can rapidly respond upon re-exposure [109]. Following antigen exposure, predicted B cell activation was observed in all constructs, and memory B cells were predicted to remain at consistent levels over time. This simulated response suggests a memory-like profile that requires experimental confirmation. These simulated immune-response patterns may support preliminary prioritisation of candidate multi-epitope constructs. Confirmation of the predicted antibody responses, cytokine profiles, and immune-cell dynamics requires further validation through in vitro and in vivo assays before biological activity can be inferred.

The expression system was prepared for initial expression by optimising the constructs for the *E. coli* host using codon optimisation. The results obtained from JCat showed CAI values of 1.0 and 0.94, and GC contents of 55.56% and 54.19% for the IA1-specific and core-predicted constructs, respectively. These values are considered satisfactory, as CAIs above 0.80 and GC contents between 30% and 70% are generally regarded as optimal for efficient gene expression, as described in a previous study [110]. Computational, immunoinformatic, and molecular dynamics analyses support prioritising the designed epitopes based on predicted antigenicity, receptor docking, immune simulation outputs, and safety-related filters. These promising in silico results warrant further validation through in vitro and in vivo studies to evaluate the constructs’ immunogenicity, safety, and functional relevance against *C. acnes*. After computational characterisation, selected constructs could be formulated with suitable adjuvants and delivery systems to enhance stability, immune cell uptake, and immunogenicity. Selected candidates could then be evaluated in vitro for purity, stability, immune recognition, and preliminary safety, followed by preclinical animal studies to assess antibody and immune cell responses, protection, and bacterial clearance [111]. Further safety, cross-reactivity, storage, and manufacturing evaluations are needed before progressing to clinical trials to confirm safety, optimal dosing, immunogenicity, and efficacy.

Another consideration of the present findings is that *C. acnes* is a common skin commensal rather than merely a pathogen. Therefore, the antigenic and epitope candidates prioritised in this study should be viewed as computationally selected targets that may be associated with acne-relevant lineages (e.g., the IA1-specific epitope) or growth conditions. The epitopes derived from the conserved core proteins may also be present in non-acne-associated or commensal strains, which represents an important limitation of the current in silico approach. Further refinements would narrow the selection of the core proteins that may be acne-related under specific strain backgrounds, host conditions, or expression contexts, while their expression or functional importance under normal skin flora conditions may be lower or context dependent. Future experimental validation should also assess strain specificity, protein expression under acne-associated versus commensal conditions, potential effects on non-acne-associated *C. acnes*, and broader impacts on the skin microbiome.

## 5. Conclusions

The number of patients struggling with acne has been increasing worldwide, accompanied by growing concern over antibiotic-resistant bacteria. There is a pressing need to discover alternative therapeutic agents. Therefore, in this study, two groups of *C. acnes* multi-epitope candidates were proposed, representing core-protein-derived and IA1-specific construct groups. These candidates were computationally predicted to generate immune-response profiles in simulation, provide broad predicted HLA population coverage, and exhibit favourable predicted safety-related profiles, including low predicted allergenicity and no significant human homology. These findings highlight the value of genomics and bioinformatics in medical research, particularly in narrowing down potential therapeutic targets for vaccine development. By enabling rapid and efficient selection of candidate proteins and epitopes, these approaches reduce complexity and time consumption, allowing researchers to more easily validate promising targets in future in vitro and in vivo studies.

## Data Availability

Data are contained within the article and Supplementary Materials.

## Supplementary Materials

The following supporting information can be downloaded at: https://www.mdpi.com/article/10.3390/biology15120933/s1.
