# Supplementary material for: Proteome-Based Antigen Screening and Multi-Epitope Design Against Cutibacterium acnes: An In Silico Study
Source: Biology (Basel). 2026 Jun 15;15(12):933. doi: 10.3390/biology15120933 (PMC13295564; doi:10.3390/biology15120933)

Supplementary Figure S1. Population coverage analysis of the predicted CTL epitopes.

The X-axis shows the predicted epitopes, and the Y-axis shows the population groups. The distinct colours indicate different levels of population coverage, and the numbers in the cells show the percentage of population coverage for each predicted CTL epitope and population group.

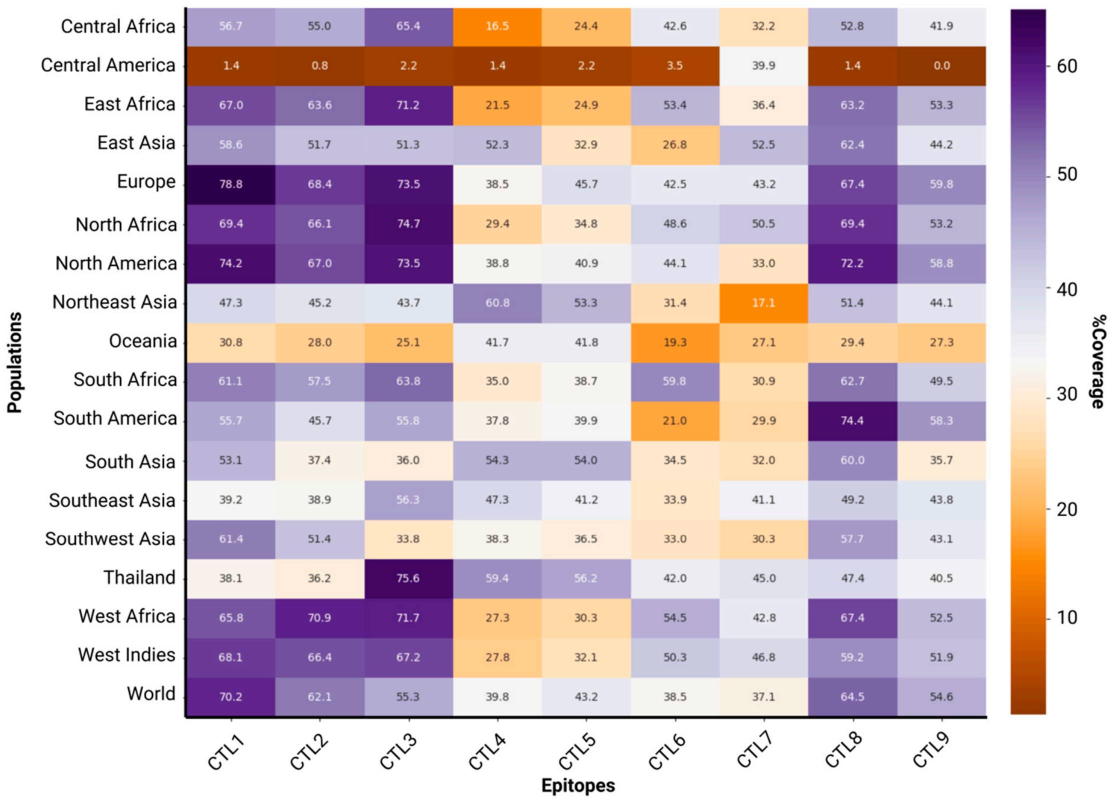

Supplementary Figure S2. Population coverage analysis of the predicted HTL epitopes.

The X-axis shows the predicted epitopes, and the Y-axis shows the population groups. The distinct colours indicate different levels of population coverage, and the numbers in the cells show the percentage of population coverage for each predicted CTL epitope and population group.

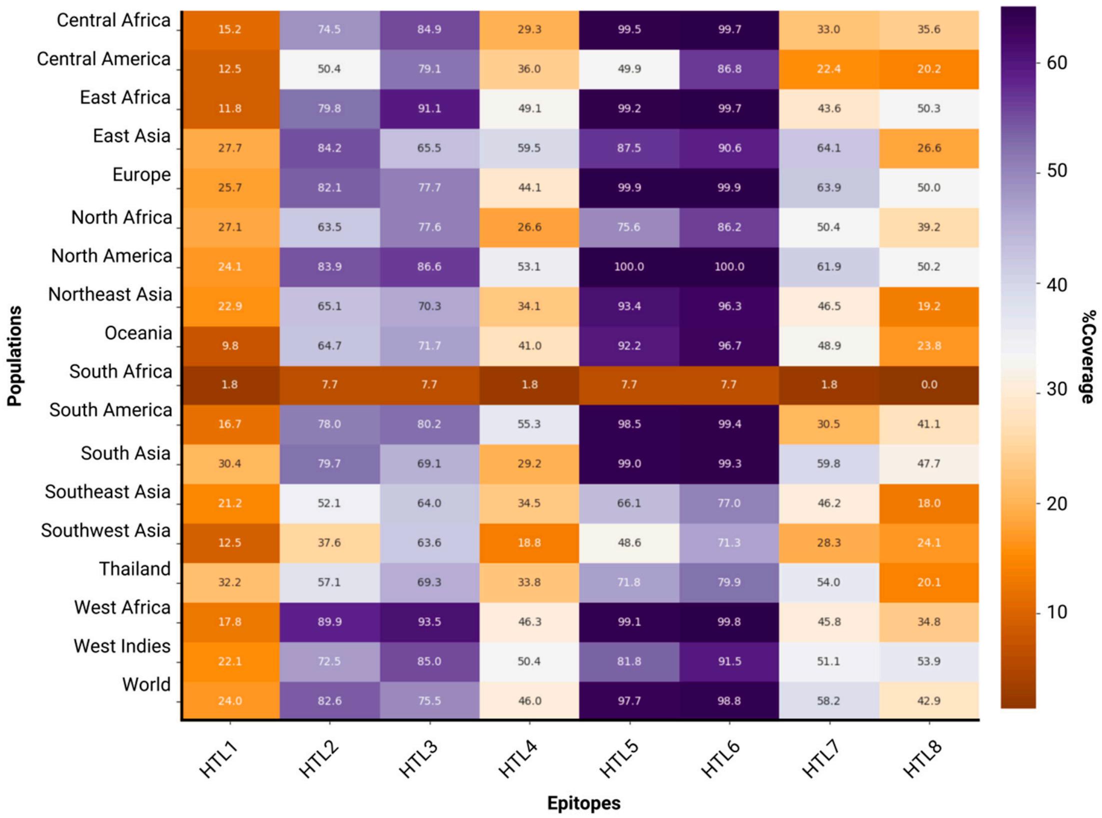

Supplementary Figure S3. Population coverage assessment of the epitope constructs.

The Y-axis represents the construct types, including core (core protein-predicted epitopes) and IA1 (IA1-specific epitope constructs). The X-axis lists the regions or countries evaluated, including Central Africa (CAF), Central America (CAM), East Africa (EAF), East Asia (EA), Europe (EU), North Africa (NAF), North America (NAM), North Asia (NA), Oceania (OC), South Africa (SAF), South America (SAM), South Asia (SA), Southeast Asia (SEA), Southwest Asia (SWA), Thailand (THA), West Africa (WAF), and the West Indies (WI). The filled colours represent the percentage of population coverage, with the numbers inside the cells showing the coverage percentage for each predicted epitope construct and population group.

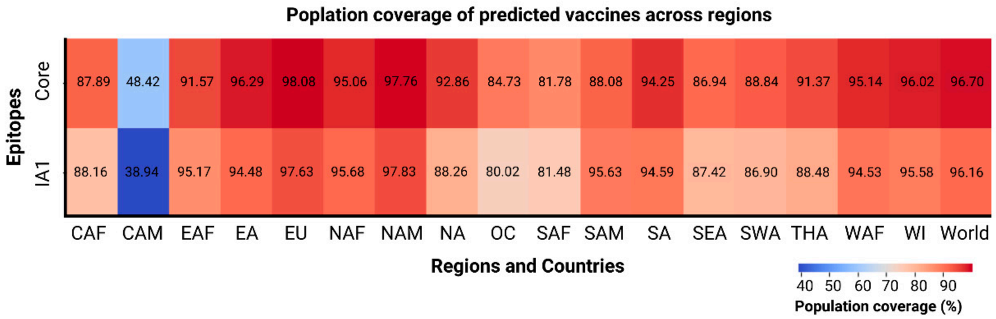

Supplementary Figure S4. Deformability and B-factor analysis of TLR2 in complex with various vaccine constructs using iMODS (Internal Coordinates Normal Mode Analysis).

Panels A–H: Deformability Analysis. These graphs show the deformability of TLR2 residues when docked with different predicted vaccine constructs. Deformability reflects the flexibility of each residue in the protein structure, where higher peaks indicate more flexible (deformable) regions. Panels A–D: IA1-specific vaccine constructs and Panels E–H: Core-predicted (MULTI) vaccine constructs. Panels I–P: B-Factor Analysis. These plots compare the B-factors (atomic displacement parameters) derived from Normal Mode Analysis (NMA) and experimental PDB data. This comparison illustrates how well the computational model aligns with experimental structural data and reflects atomic mobility. Panels I–L: IA1-specific vaccine constructs and Panels M–P: Core-predicted (MULTI) vaccine constructs

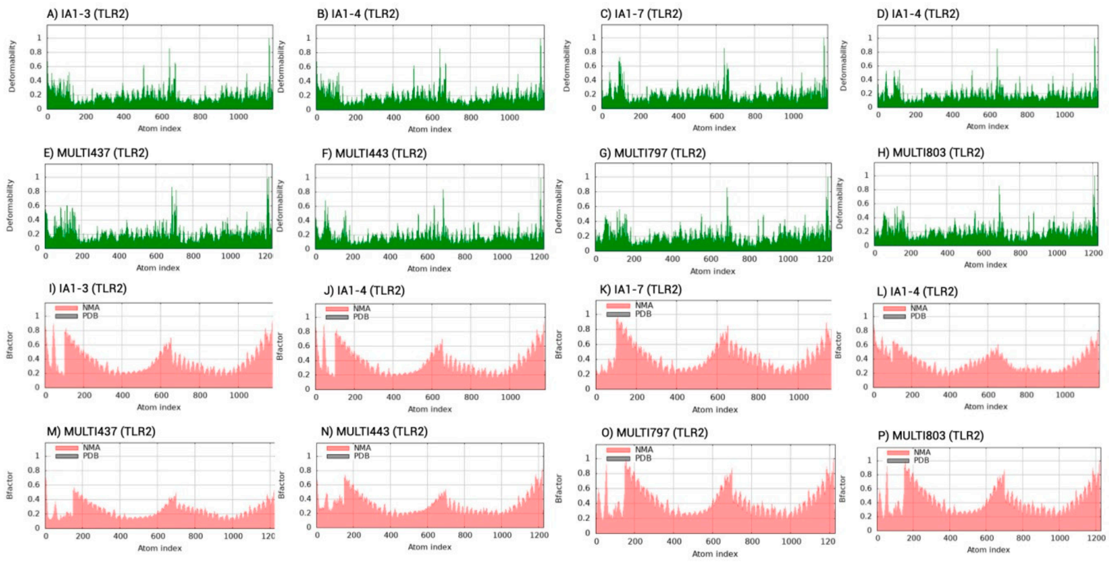

Supplementary Figure S5. Deformability and B-factor analysis of TLR4 in complex with various vaccine constructs using iMODS (Internal Coordinates Normal Mode Analysis).

Panels A–H: Deformability Analysis. These graphs show the deformability of TLR4 residues when docked with different predicted vaccine constructs. Deformability reflects the flexibility of each residue in the protein structure, where higher peaks indicate more flexible (deformable) regions. Panels A–D: IA1-specific vaccine constructs and Panels E–H: Core-predicted (MULTI) vaccine constructs. Panels I–P: B-Factor Analysis. These plots compare the B-factors (atomic displacement parameters) derived from Normal Mode Analysis (NMA) and experimental PDB data. This comparison illustrates how well the computational model aligns with experimental structural data and reflects atomic mobility. Panels I–L: IA1-specific vaccine constructs and Panels M–P: Core-predicted (MULTI) vaccine constructs

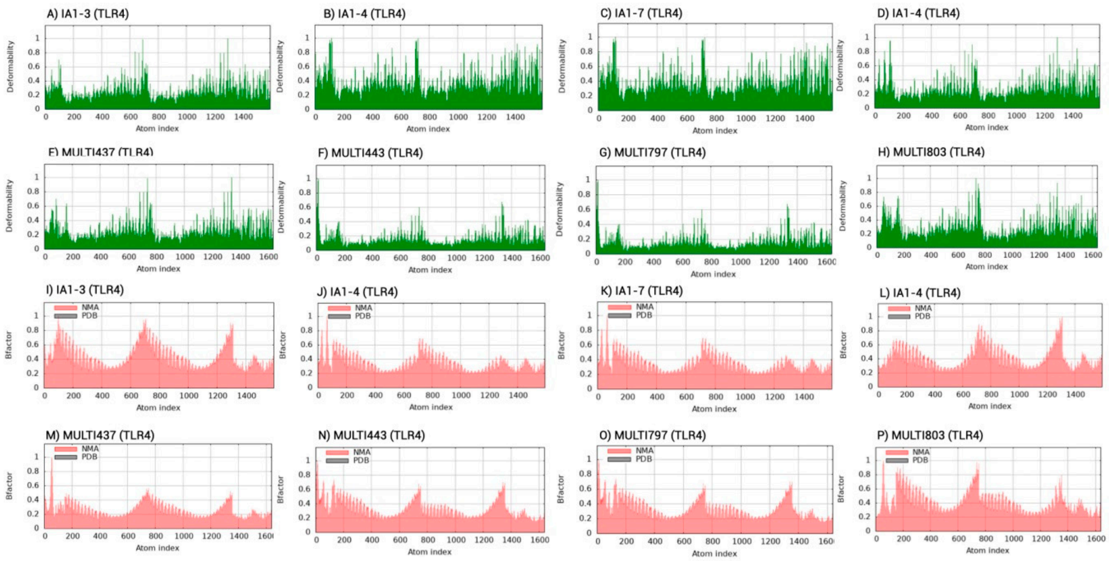

Supplementary Figure S6. Eigenvalue and variance analysis of TLR2 in complex with various vaccine constructs using iMODS (Internal Coordinates Normal Mode Analysis).

Panels A–H: Eigenvalue Analysis. These plots show the eigenvalues associated with each normal mode of motion for TLR2 when docked with different vaccine constructs. A lower eigenvalue corresponds to greater structural flexibility and easier deformation, indicating less energy is required for structural motion. Panels A–D: IA1-specific vaccine constructs. Panels E–H**:** Core-predicted (MULTI) vaccine constructs. Each graph displays the eigenvalue corresponding to the first mode, which is typically the most functionally relevant large-scale motion.

Panels I–P: Variance Analysis. These bar graphs represent the percentage of variance explained by each of the first 20 normal modes. Higher cumulative variance indicates that fewer modes are needed to describe most of the protein's motion, which is important for understanding the dominant conformational changes**.** Panels I–L**:** IA1-specific vaccine constructs**.** Panels M–P: Core-predicted (MULTI) vaccine constructs

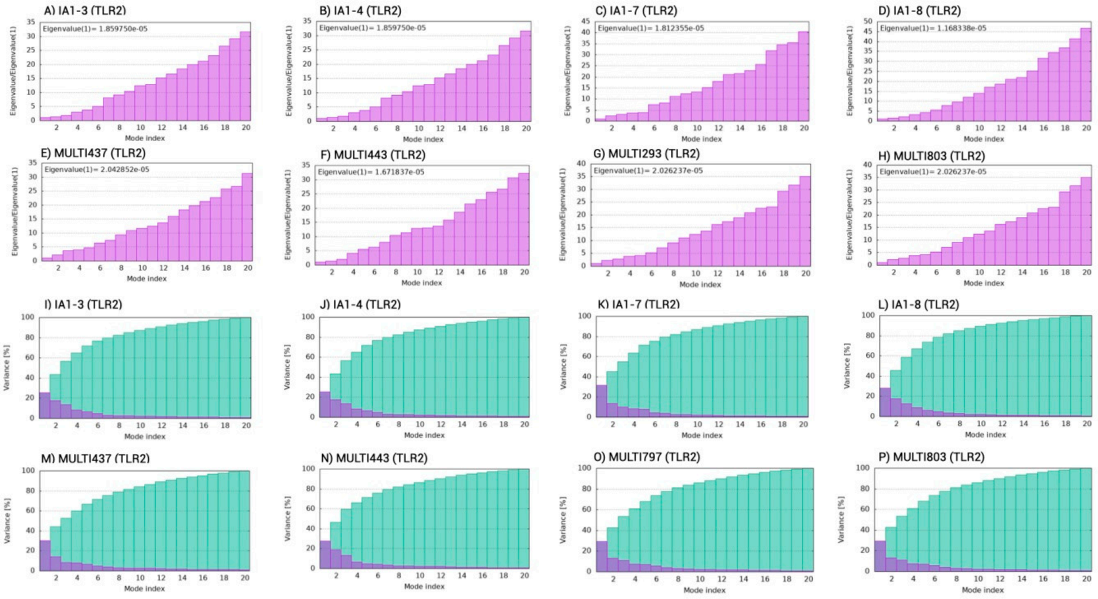

Supplementary Figure S7. Eigenvalue and variance analysis of TLR4 in complex with various vaccine constructs using iMODS (Internal Coordinates Normal Mode Analysis).

Panels A–H: Eigenvalue Analysis. These plots show the eigenvalues associated with each normal mode of motion for TLR4 when docked with different vaccine constructs. A lower eigenvalue corresponds to greater structural flexibility and easier deformation, indicating less energy is required for structural motion. Panels A–D: IA1-specific vaccine constructs. Panels E–H**:** Core-predicted (MULTI) vaccine constructs. Each graph displays the eigenvalue corresponding to the first mode, which is typically the most functionally relevant large-scale motion.

Panels I–P: Variance Analysis. These bar graphs represent the percentage of variance explained by each of the first 20 normal modes. Higher cumulative variance indicates that fewer modes are needed to describe most of the protein's motion, which is important for understanding the dominant conformational changes**.** Panels I–L**:** IA1-specific vaccine constructs**.** Panels M–P: Core-predicted (MULTI) vaccine constructs

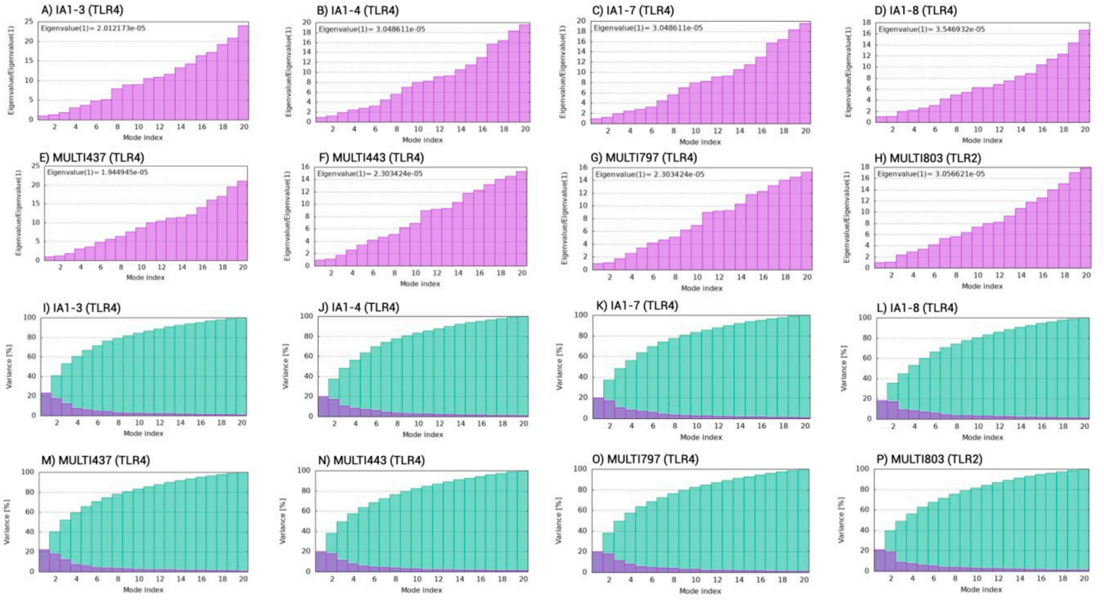

***Supplementary Figure S*8**. Covariance and elastic network analysis of TLR2 in complex with various vaccine constructs using iMODS.

Panels A–H: Covariance Matrix Analysis. These plots represent the covariance of atomic fluctuations between residue pairs in TLR2 when docked with different vaccine constructs. Red regions indicate correlated motion (residues move in the same direction).Blue regions indicate anti-correlated motion (residues move in opposite directions). This matrix helps identify regions of coordinated flexibility that may play a role in structural transitions. Panels A–D: IA1-specific vaccine constructs and Panels E–H: Core-predicted (MULTI) vaccine constructs.

Panels I–P: Elastic Network Model (ENM) Analysis. These graphs show the elastic network representation of TLR2. Each dot corresponds to a spring connecting two atoms (nodes), representing stiffness and interaction strength between them. The denser and darker regions along the diagonal represent strong local interactions, often within secondary structure elements. Off-diagonal patterns may indicate long-range interactions critical for allosteric communication. Panels I–L: IA1-specific vaccine constructs **and** Panels M–P: Core-predicted (MULTI) vaccine constructs

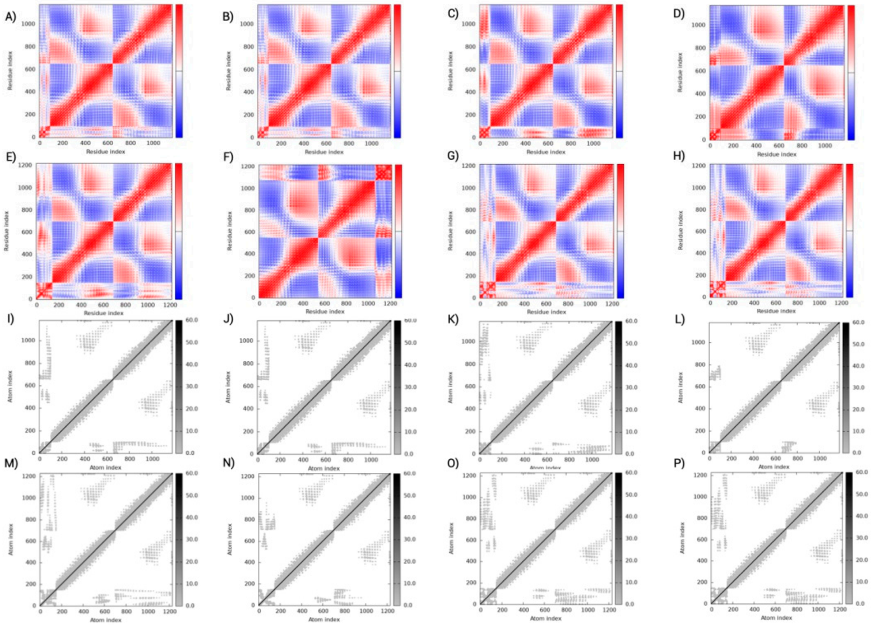

*Supplementary Figure S*9. Covariance and elastic network analysis of TLR4 in complex with various vaccine constructs using iMODS.

Panels A–H: Covariance Matrix Analysis. These plots represent the covariance of atomic fluctuations between residue pairs in TLR4 when docked with different vaccine constructs. Red regions indicate correlated motion (residues move in the same direction).Blue regions indicate anti-correlated motion (residues move in opposite directions). This matrix helps identify regions of coordinated flexibility that may play a role in structural transitions. Panels A–D: IA1-specific vaccine constructs and Panels E–H: Core-predicted (MULTI) vaccine constructs.

Panels I–P: Elastic Network Model (ENM) Analysis. These graphs show the elastic network representation of TLR4. Each dot corresponds to a spring connecting two atoms (nodes), representing stiffness and interaction strength between them. The denser and darker regions along the diagonal represent strong local interactions, often within secondary structure elements. Off-diagonal patterns may indicate long-range interactions critical for allosteric communication. Panels I–L: IA1-specific vaccine constructs **and** Panels M–P: Core-predicted (MULTI) vaccine constructs

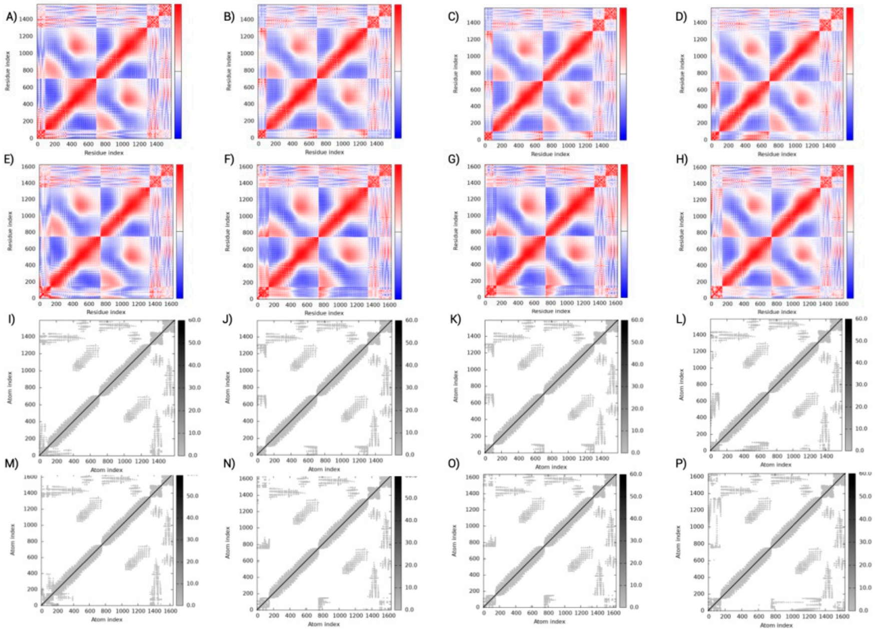

Supplementary Figure S10. Simulation of antigen and antibody titer dynamics following epitope administration. Panels A–D show the IA1 *C. acnes*-specific predicted epitope constructs, while panels E–H depict the *C. acnes* core predicted multi-epitope constructs and their associated antibody titer dynamics. The left Y-axis represents antigen concentration (count/mL), while the right Y-axis indicates antibody titers, including IgM, IgG1, and IgG2. Antibody titers refer to the highest dilution of a blood serum sample at which antibodies remain detectable against a specific antigen; higher titers suggest a stronger immune response. The X-axis shows the time course of immune activity. Each line colour represents a different component of the immune response: antigen (black), total IgM+IgG (yellow), IgM (green), total IgG1+IgG2 (cyan), IgG1 (purple), and IgG2 (red).

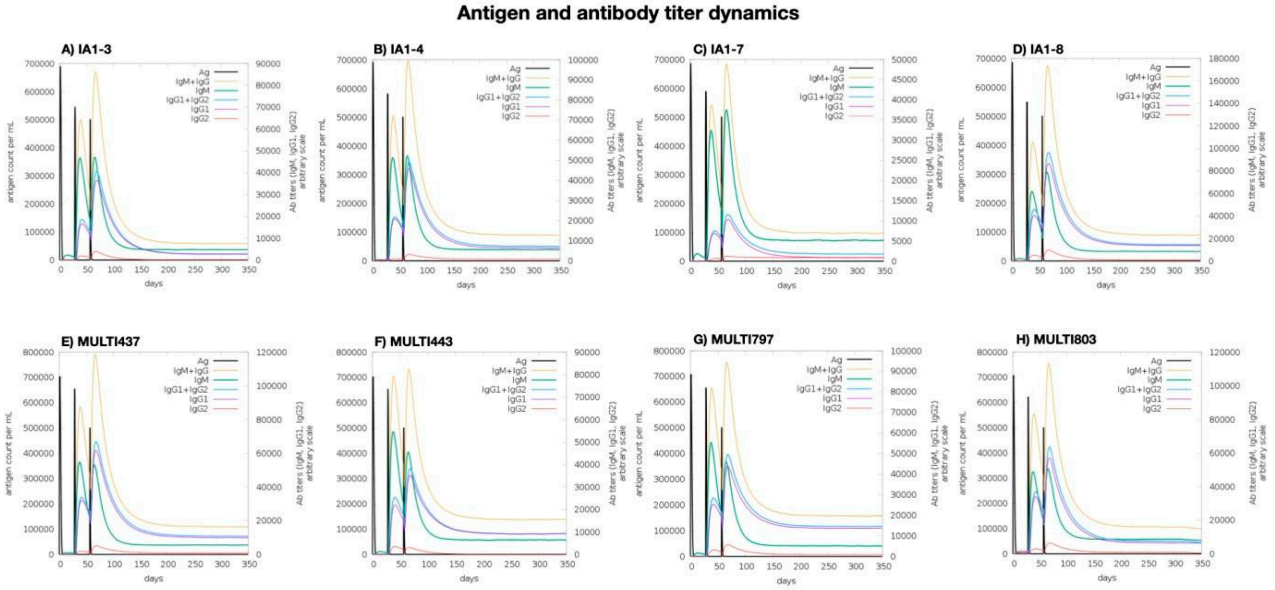

Supplementary Figure S11. The simulation of cytokine secretion profile following epitope administration. Panels A–D show the IA1 *C. acnes*-specific predicted epitope constructs, while panels E–H depict the *C. acnes* core predicted multi-epitope constructs and their associated cytokine secretion. The Y-axis shows the concentration of cytokines (ng/mL) at the time (days) of administration, as shown on the X-axis. Colour-coded curves represent different immune signalling molecules. The smaller box inside the graph represents IL-2 levels (ng/mL) at the time points indicated on the X-axis, with the Y-axis showing the time of vaccination. "D" tracks the intensity of immune system alertness due to perceived "danger" signals.

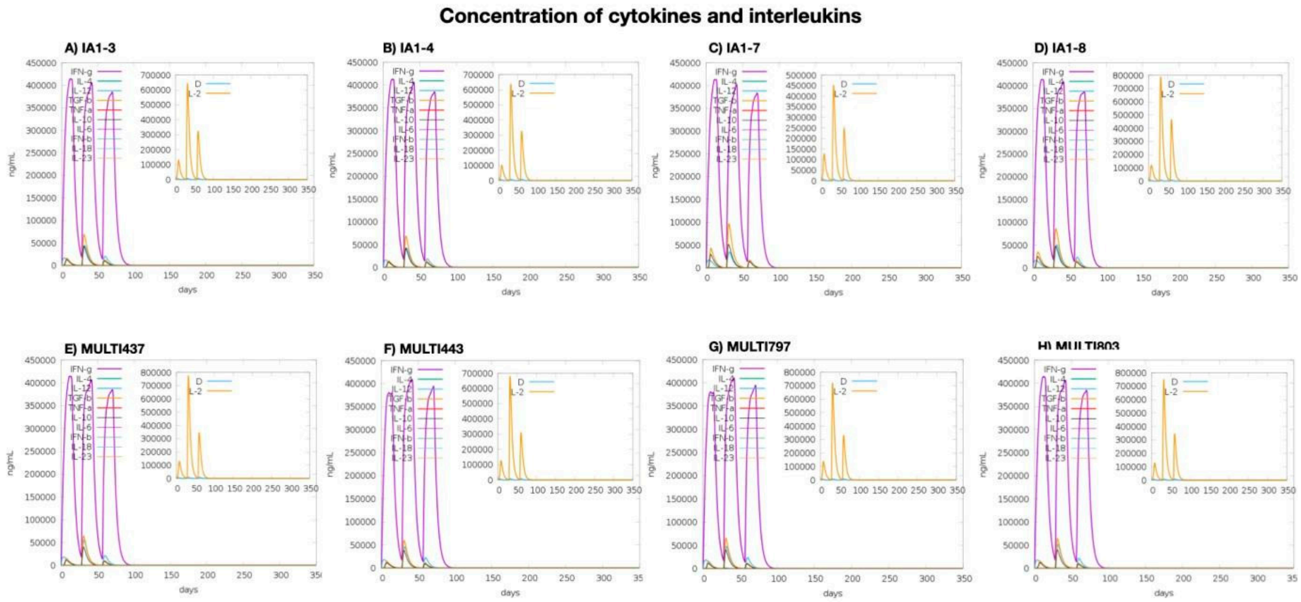

Supplementary Figure S12. Simulation of the B cell population profile following epitope administration. Panels A–D show the IA1 *C. acnes*-specific predicted epitope constructs, while panels E–H depict the *C. acnes* core predicted multi-epitope constructs and their associated B cell population responses. The left Y-axis represents the quantities of various B cell subtypes, excluding memory B cells, which are shown on the right Y-axis. The X-axis indicates the time in days after post-epitope administration. Colour-coded curves represent different B cell subpopulations.

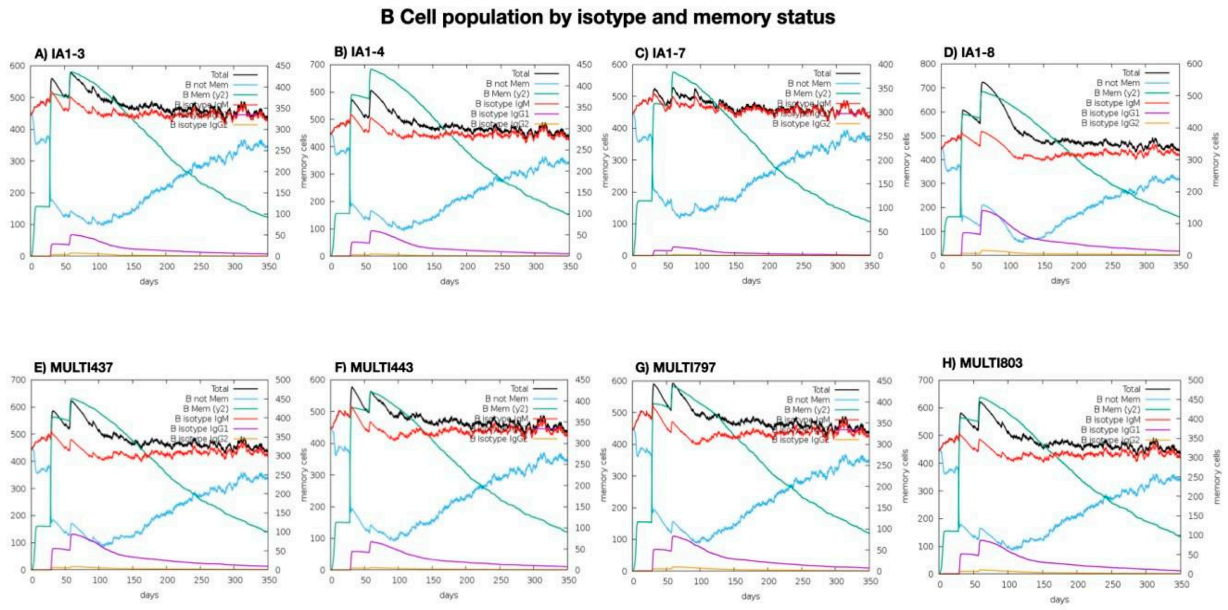

Supplementary Figure S13. Vector constructs for the initial expression system of predicted epitope constructs. Both vectors were constructed based on the pET-28a(+) vector. The insertion target for both epitope constructs is represented by the red fragments, located between the BamHI and Xhol restriction sites.

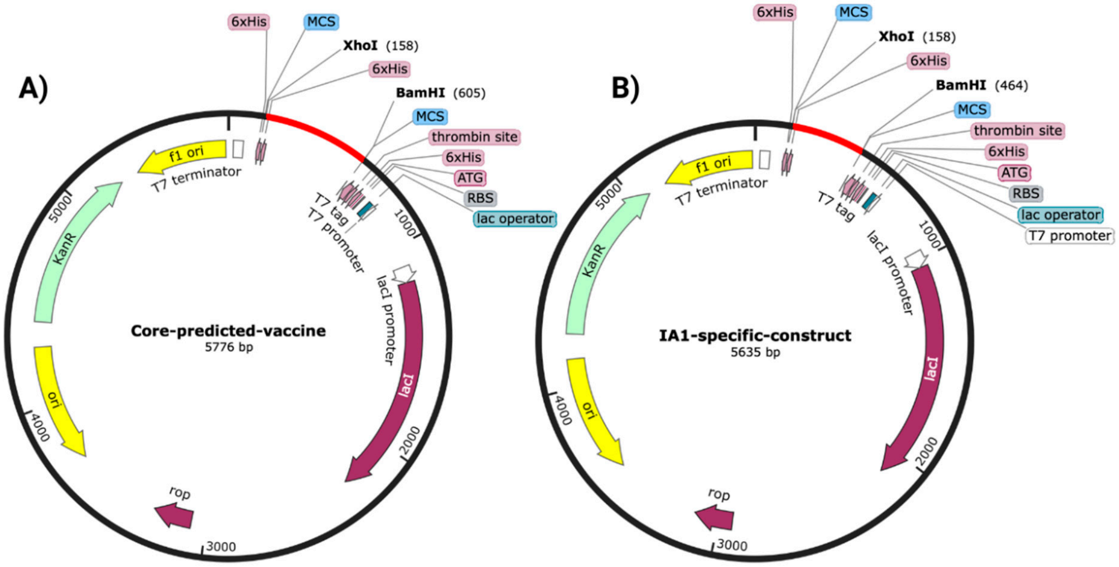

Supplement: Supplementary file 1 [file biology-15-00933-s001.zip › biology-4239792_Supplementary_Figures_Revised.pdf]
